# Disentangling the Effects of Water Stress on Carbon Acquisition, Vegetative Growth, and Fruit Quality of Peach Trees by Means of the QualiTree Model

**DOI:** 10.3389/fpls.2018.00003

**Published:** 2018-01-24

**Authors:** Mitra Rahmati, José M. Mirás-Avalos, Pierre Valsesia, Françoise Lescourret, Michel Génard, Gholam H. Davarynejad, Mohammad Bannayan, Majid Azizi, Gilles Vercambre

**Affiliations:** ^1^UR 1115, Plantes et Systèmes de Culture Horticoles, Institut National de la Recherche Agronomique, Avignon, France; ^2^Faculty of Agriculture, Ferdowsi University of Mashhad, Mashhad, Iran

**Keywords:** carbon allocation, drought, energy balance, light interception, photosynthesis, growth, process-based model, *Prunus persica* L.

## Abstract

Climate change projections predict warmer and drier conditions. In general, moderate to severe water stress reduce plant vegetative growth and leaf photosynthesis. However, vegetative and reproductive growths show different sensitivities to water deficit. In fruit trees, water restrictions may have serious implications not only on tree growth and yield, but also on fruit quality, which might be improved. Therefore, it is of paramount importance to understand the complex interrelations among the physiological processes involved in within-tree carbon acquisition and allocation, water uptake and transpiration, organ growth, and fruit composition when affected by water stress. This can be studied using process-based models of plant functioning, which allow assessing the sensitivity of various physiological processes to water deficit and their relative impact on vegetative growth and fruit quality. In the current study, an existing fruit-tree model (QualiTree) was adapted for describing the water stress effects on peach (*Prunus persica* L. Batsch) vegetative growth, fruit size and composition. First, an energy balance calculation at the fruit-bearing shoot level and a water transfer formalization within the plant were integrated into the model. Next, a reduction function of vegetative growth according to tree water status was added to QualiTree. Then, the model was parameterized and calibrated for a late-maturing peach cultivar (“Elberta”) under semi-arid conditions, and for three different irrigation practices. Simulated vegetative and fruit growth variability over time was consistent with observed data. Sugar concentrations in fruit flesh were well simulated. Finally, QualiTree allowed for determining the relative importance of photosynthesis and vegetative growth reduction on carbon acquisition, plant growth and fruit quality under water constrains. According to simulations, water deficit impacted vegetative growth first through a direct effect on its sink strength, and; secondly, through an indirect reducing effect on photosynthesis. Fruit composition was moderately affected by water stress. The enhancements performed in the model broadened its predictive capabilities and proved that QualiTree allows for a better understanding of the water stress effects on fruit-tree functioning and might be useful for designing innovative horticultural practices in a changing climate scenario.

## Introduction

The occurrence of extreme events is predicted to increase in intensity, frequency, and geographic extent due to climate change (IPCC, 2014). As a consequence, agriculture will face increasing periods of drought and water deficit is expected to exert adverse impacts upon plant growth and productivity (Shao et al., 2008). This decreased water availability and increased evaporative demand would impair carbon gain by photosynthesis and expansive growth of vegetative and reproductive organs (Tardieu et al., 2014). Nevertheless, growth and photosynthesis respond differently to environmental conditions. In fact, expansive growth is the most sensitive physiological response related directly to water deficit (Hsiao, 1973) while photosynthetic limitation associated to stomatal closure occurs latter (Ings et al., 2013).

Water stress reduce plant vegetative growth and leaf photosynthesis (Hsiao, 1973; Harrison et al., 1989; Berman and DeJong, 1997; Chaves et al., 2002; Lampinen et al., 2004; Bryla et al., 2005; Girona et al., 2005; Shao et al., 2008; Muller et al., 2011; Tardieu, 2013; Rahmati et al., 2015a) whereas a low water deficit could have no impact on photosynthesis yield and growth (Álvarez et al., 2009; Forey et al., 2016) or even have a beneficial effect if the water deficit is temporary and followed by rewatering (Zhao et al., 2015; Yi et al., 2016). Moreover, management practices such as regulated deficit irrigation (RDI) are based on inducing a mild water stress to the plants that stops vegetative growth but allows for maintaining or even increase yield while saving water (Scott Johnson and Handley, 2000).

Vegetative and reproductive growth often occur simultaneously in fruit trees, which under stressful environmental conditions may result in a higher competition for carbohydrates and water resources among organs (Grossman and DeJong, 1995). However, previous research showed that vegetative growth can be more reduced by water deficit than fruit growth (Yuan et al., 2009). A slowdown of shoot growth rate in response to a decrease of stem water potential has been observed in peach and olive (Berman and DeJong, 1997; Solari and DeJong, 2006; Gómez-del-Campo, 2013). Similarly, fruit fresh weight is reduced by water stress (Berman and DeJong, 1996; Bryla et al., 2005; Mirás-Avalos et al., 2016); however, fruit sink strength is more resilient, as shown by the maintenance of fruit dry mass under water constrains (Berman and DeJong, 1996). In addition, the alterations on plant metabolism caused by water stress exert a significant effect not only on tree growth but also on fruit quality, an important issue for fruit production and retailing (Codron et al., 2005), which involves a set of traits including fruit size and chemical composition that result from many processes at both the plant and organ levels (Génard et al., 2009).

Due to the complexity of all these phenomena, a global quantification of the effects of water stress on tree physiological processes and their interrelations has not been previously undertaken. Nevertheless, under the changing climate scenario due to global warming, it is interesting to assess whether the sink strength (growth) or the source activity (photosynthesis), as affected by water stress, exert a differential influence on plant vegetative and reproductive growth, as well as on fruit composition in order to understand plant behavior, which will provide a framework for developing sustainable agricultural practices.

In this context, process-based simulation models emerge as useful tools for improving our understanding of the complex linked processes that control tree growth, fruit size, and composition at different organizational levels (Martre et al., 2011). In fact, when the studied system is complex (e.g., a plant), simulation models allow for analyzing how the system works (in terms of interacting processes) under the control of environmental, genetic, and plant factors. The model offers a theory describing how the components of the system causally interact to produce a given outcome. Thus, simulations provide a virtual representation of relevant aspects of the real system under investigation (Peck, 2004). In the case of fruit trees, a number of models for simulating plant functioning are available for different species such as apple (Costes et al., 2008), kiwi (Cieslak et al., 2011), and peach (Allen et al., 2005). However, few models account for the effects of both water and carbon availability on sink and source organs of fruit trees under stressful environments. One exception is the L-PEACH model where the growth of individual organs is not only influenced by carbon partitioning, but also by stem water potential (Da Silva et al., 2011). However, this approach does not consider fruit quality attributes other than size.

In contrast, the QualiTree model (Lescourret et al., 2011) combines physiological and agronomic viewpoints for describing carbon allocation within the tree, vegetative, and fruit growth distributions, and the development of fruit quality, which is its main advantage with respect to other fruit-tree models. QualiTree is a “source-sink” plant model that integrates an empirical function to simulate the impact of distances on assimilate allocation in a simplified representation of tree architecture. A previous version of this model described the within-tree variability of growth and fruit quality traits and was successfully calibrated and validated with experimental data (Mirás-Avalos et al., 2011, 2013a,b). In this version, water deficit affected vegetative growth indirectly through a reduced leaf photosynthesis and hence decreased production of carbohydrates but this was not enough to reproduce the strong decrease in vegetative growth observed in the field and caused by water stress. These previous articles allowed for describing the main principles that constitute the base of QualiTree and for parameterizing, calibrating and validating the model for four peach cultivars with different growing cycles. However, some weak points were detected in the model, such as the lack of a description of the variability of water status within the tree and the lack of a function that accounts for the direct effects of water stress on vegetative growth. The current work describes some improvements that allowed us to address these shortcomings of the previous model. Furthermore, the new implementations permitted us to differentiate the level of incidence of water status on photosynthesis and sink strength.

Peach trees (*Prunus persica* L. Bastch) are widely grown in Mediterranean countries usually under water shortage conditions. Water stress at stage III of fruit development, which is a period of rapid growth of fruit skin (exocarp) and flesh (mesocarp), limits fruit size in peach (Berman and DeJong, 1996), and may impede attaining marketable fruit size. In contrast, other fruit quality criteria could be positively affected by water constraints, since increases in total soluble solids content (Crisosto et al., 1994; Lopez et al., 2011) and reductions in organic acid concentrations (Thakur and Singh, 2012) have been observed. Due to the great amount of experimental evidence of the effects of water stress on peach vegetative and fruit growth, this species represents a good biological model for analyzing the influence of water stress on plant vegetative and fruit growth.

The purposes of the current study were to (i) improve the virtual fruit-tree model QualiTree by implementing a new module for energy balance calculation and water transfer within the tree and by including a direct effect of plant water status on vegetative growth; (ii) validate the model using experimental data (vegetative and reproductive growth and fruit quality) from a peach cultivar grown under semi-arid conditions; (iii) and assess the relative importance of water constrains on the reduction in source activity (photosynthesis) and sink strength (growth).

## Materials and methods

### Overview of the QualiTree model

QualiTree (Lescourret et al., 2011) is a generic fruit-tree model that considers the tree as a set of objects: some compartments viewed globally such as old wood (trunk and branches), water sprouts, and roots (coarse and fine), and some viewed in more detail and placed in the tree architecture, such as fruiting units (FU). The FU are composed of fruits, leafy shoots, and stem wood. The tree is virtually regarded as a collection of FU connected to old wood within an explicit architecture (topology and geometry). A simple module for estimating canopy radiation interception over the growing season was used to simulate the photosynthesis on each FU (Mirás-Avalos et al., 2011).

The growth in dry mass of tree organs is represented in QualiTree through a carbon-supply approach, allocation rules (priority sequence between processes—e.g., maintenance, then growth—and organs—e.g., leafy shoot growth, then fruit growth; use of reserve as buffers; passive carbon storage), and equations of carbon assimilation (based on leaf area for photosynthesis) and growth requirements (demands). These equations are taken mainly from the FU carbon model by Lescourret et al. (1998).

In order to restore within-tree carbon balance, QualiTree employs two main principles. First, a root-shoot functional balance is used with a target ratio at equilibrium, according to the coordination theory (Reynolds and Chen, 1996). Second, carbohydrates are allocated among the tree organs depending on the supply of the donor, the demands of the recipient, and a decreasing effect of geometric distance between donor and recipient using a negative power law (Lescourret et al., 2011).

Moreover, QualiTree represents the development of several fruit quality traits including size, flesh dry matter content and concentrations of various sugars. In QualiTree, fruit sugar content is described by a set of differential equations (Génard et al., 2003) that accounts for the three processes involved in the development of soluble sugars (sucrose, glucose, fructose, and sorbitol) within the fruit: sugar importation, metabolism and water dilution (Dai et al., 2016).

The effects of environmental conditions and agricultural practices on tree growth are included in QualiTree. From an initial state of the tree, QualiTree runs on a daily time-step (hourly for photosynthesis and water balance), from bloom or after bloom until the end of the fruit growing season.

A comprehensive description of QualiTree can be found in Lescourret et al. (2011, 2016). Parameters and validations for several peach cultivars are reported in Mirás-Avalos et al. (2011, 2013a,b). A list of QualiTree inputs and outputs is provided in Supplementary Tables 1, 2.

### Improvements and new implementations in QualiTree

#### Light interception calculation

In a previous version of QualiTree, a simple radiation interception model was implemented (Mirás-Avalos et al., 2011). Briefly, this module predicts radiation interception by a tree based on the works of Charles-Edwards and Thornley (1973) and de Pury and Farquhar (1997). The tree is within an orchard and its canopy is represented by simple geometric shapes (ellipsoids). In the current study, the calculation of the radiation balance has been integrated into this module.

For each FU, the absorbed radiation is split in three wavebands: Photosynthetically Active Radiation (PAR), Near Infrared Radiation (NIR), and Thermal Infrared Radiation (TIR). Leaves have specific properties (absorption, reflection, transmission) for these three wavebands. In the TIR, incident radiation is related to sky, soil, and leaf emissivity and their respective temperatures. The net radiation (*Rn*_*i*_) can be defined as:

(1) $$Rn_{i}=PAR_{i}+NIR_{i}+TIR_{i}-2\sigma {T_{leaf}}_{i}^{4}A_{i}$$

where *i* is the index of a given FU, the last term accounts for the emitted radiation of the considered FU according to its temperature (*T*_*leaf*_), leaf surface (*A*_*i*_), and the Stefan-Boltzmann constant (σ).

#### Temperature, transpiration, and water potential calculation within the tree

Linked to this radiation model, an energy balance has been implemented into QualiTree:

(2) $$Rn_{i}=S_{i}+\lambda E_{i}$$

where *S*_*i*_ and λ*E*_*i*_ are the sensible and latent heat fluxes, respectively. These fluxes are computed following the approach described by Sinoquet et al. (2001). Leaf boundary conductance encompasses a free component associated to the difference in leaf and air temperatures and a forced component related to wind speed around the leaf (Daudet et al., 1999). Stomatal conductance is expressed through the multiplicative empirical model of Jarvis (1976):

(3) $$g_{s}=g_{smax}f_{1}\left(PAR\right)f_{2}\left(VPD\right)f_{3}\left(T_{leaf}\right)f_{4}\left(\psi_{leaf}\right)$$

Therefore, the stomatal conductance (*g*_*s*_) is related to a maximum value (*g*_*smax*_) decreased by limiting functions associated with the incident PAR on the leaf, vapor pressure deficit (VPD), leaf temperature (*T*_*leaf*_), and leaf water potential (Ψ_*leaf*_). By solving the energy balance (Equations 1 and 2), leaf temperature is estimated. The model separates the energy balance of sunlit and shaded areas, since differences in leaf temperature are expected (Sinoquet et al., 2001). Net radiation is sensitive to leaf temperature through the leaf emitted thermal radiation, and the energy balance is sensitive to leaf temperature and to leaf water potential through boundary and stomatal conductances.

Water flow within plant three-dimensional architecture is described by laws that connect the different axes through their hydraulic conductivity (m^4^ s^−1^ MPa^−1^) and by the water potential decrease along the axis (Fiscus, 1975). Hydraulic conductivity is assumed to vary according to axis diameter (Tyree, 1988; Vercambre et al., 2002). The boundary condition is imposed at the root collar through diurnal variation in the xylem water potential.

**Figure 1:**
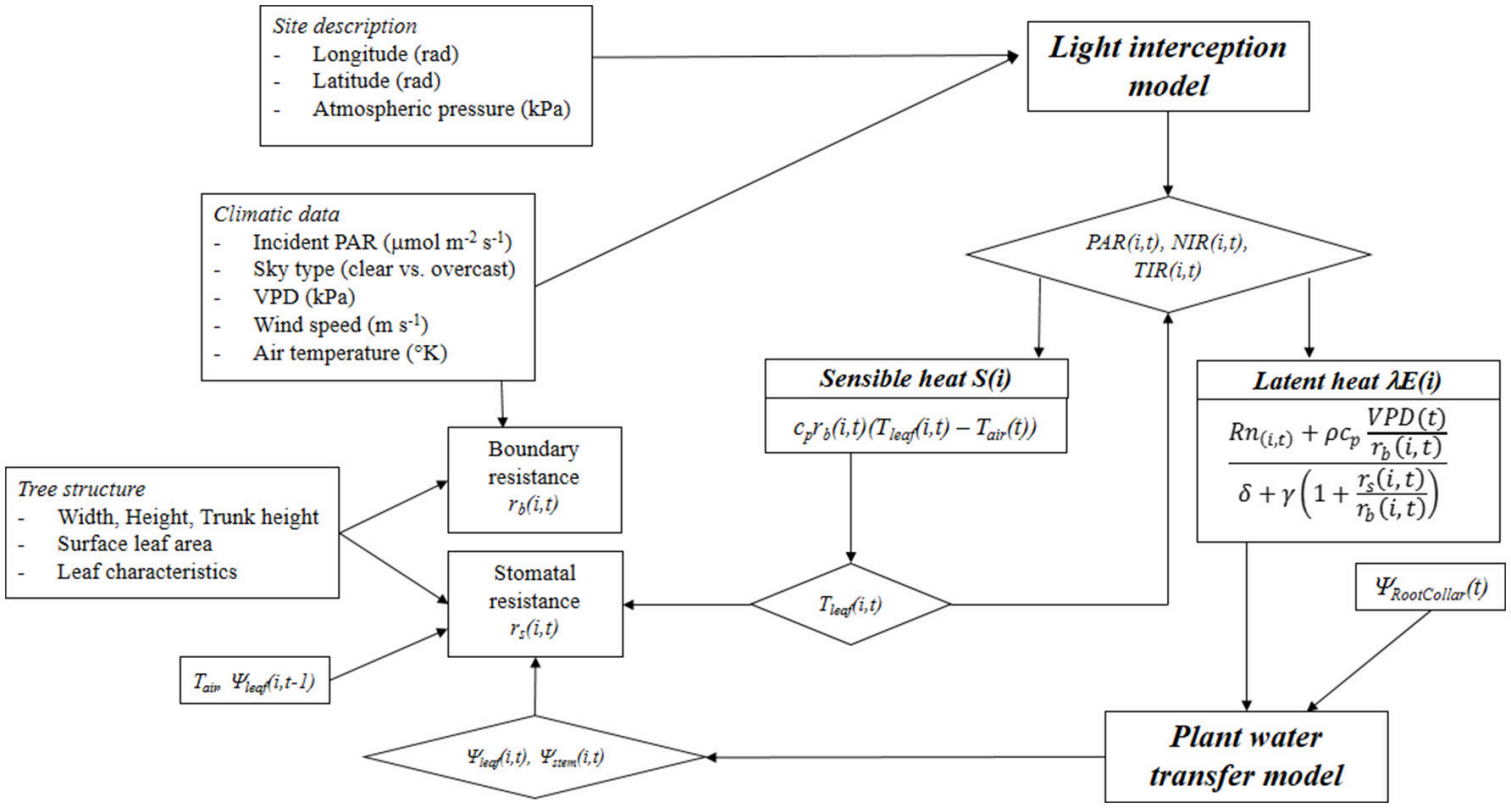
Schematic representation of the integrated light interception—radiative balance—plant water transfer model. PAR, Photosynthetically Active Radiation; NIR, Near Infrared Radiation; TIR, Thermal Infrared Radiation; *c*_*p*_ is specific heat of air at constant pressure; *r*_*b*_ and *r*_*s*_ are the boundary and stomatal resistances, respectively; *T*_*leaf*_ and *T*_*air*_ are the leaf and air temperatures, respectively; *Rn* is the net radiation; ρ is the air density; VPD, Vapor Pressure Deficit; δ is the Stefan-Boltzman constant; γ is the psychometric constant; and Ψ_*leaf*_, Ψ_*stem*_, and Ψ_*RootCollar*_ are leaf, stem, and collar water potentials, respectively.

Leaf temperature and water potential are estimated through a nested iterative process. Leaf water potential is an output of the energy balance model linked to the hydraulic transfer model. Finally, a spatially explicit estimation of leaf temperature, transpiration and water potential distribution within the tree canopy is obtained on an hourly basis (Figure 1).

#### Representation of water deficit effect on source and sink activities

In order to account for the effect of water deficit on carbon sources, the light-saturated leaf photosynthesis rate is estimated as a function of Ψ_*leaf*_ (Ben Mimoun et al., 1999) through the Harris equation (Harrison et al., 1989):

(4) $$P_{max,i}=P_{max0,i}\left(1-exp^{\left(B_{h}\left(A_{h}+\frac{1}{\psi_{leaf,i}}\right)\right)}\right)$$

Where, for a given FU, *P*_*max, i*_ is the potential light-saturated photosynthesis rate (mmol CO_2_ m^−2^ s^−1^), *P*_*max*0, *i*_ is the maximal photosynthesis rate in absence of water stress (mmol CO_2_ m^−2^ s^−1^), *A*_*h*_ and *B*_*h*_ are specific parameters, and Ψ_*leaf, i*_ is the leaf water potential. Therefore, when the tree starts to experience water stress, photosynthesis is reduced and, indirectly, carbon allocation and growth are impacted.

#### Representation of water deficit effect on potential vegetative growth

In order to simulate the effect of water deficit on tree growth, a function depending on the leaf water potential was added to the equation describing the potential growth in dry mass of vegetative organs:

(5) $$\frac{\Delta DM_{i}}{\Delta dd}=RGR_{i}^{ini}Im^{-1}f\left(dd\right)DM_{i}\left(1-\frac{DM_{i}}{Im^{-1}DM_{i}^{max}}\right)f\left(\psi_{leaf,i}\right)$$

where *i* is the index of a given FU, *RGR*^*ini*^ (dd^−1^) is the initial relative growth rate of the leafy shoot or water sprout, *DM*^*max*^ (g) is the maximum dry mass of either leafy shoot per FU or water sprout per tree, *Im* (dimensionless) is the imbalance between leafy shoots and fine root masses, and *f(dd)* is defined as:

(6) $$\begin{array}{l} f(dd)=0\;if\;dd<dd_{0}\\ f(dd)=1\;if\;dd_{0}\;\leq \;dd<dd_{min}\\ f\left(dd\right)=\frac{dd_{max}-dd}{dd_{max}-dd_{min}}\;if\;dd_{min}\leq dd<dd_{max}\\ f(dd)=0\;if\;dd\;\geq \;ddmax \end{array}$$

where *dd*_0_, *dd*_*min*_, and *dd*_*max*_ are organ-specific parameters (degree-day).

In Equation (5), the growth is limited by *f(*Ψ_*leaf, i*_*)*, which varies between 0 and 1:

(7) $$\begin{array}{l} \;f(\psi_{leaf,i})=0\;if\;\psi_{leaf,i}<\psi_{min}\\ f\left(\psi_{leaf,i}\right)=\frac{\psi_{leaf,i}-\psi_{min}}{\psi_{max}-\psi_{min}}\;if\;\psi_{min}\leq \psi_{leaf,i}<\psi_{max}\\ \;f(\psi_{leaf,i})=1\;if\;\psi_{leaf,i}\;\geq \;\psi_{max} \end{array}$$

where Ψ_*min*_ and Ψ_*max*_ are parameters of the model.

#### Effect of plant water potential on fruit growth

This module was adapted from the biophysical model of fruit growth originally developed by Fishman and Génard (1998) and modified by Génard et al. (2010), which describes the water entering the fruit by peduncular flow and leaves via transpiration. Therefore, the fruit volume varies as:

(8) $$\frac{dV}{dt}=A\;L\;\left(\psi_{stem}-\psi_{f}\right)-T$$

where *V* is fruit volume, *t* is time, *A* is the vascular network area, *L* is the hydraulic conductivity of vascular network membranes, Ψ_*stem*_ and Ψ_*f*_ are the stem and fruit water potentials, Ψ_*f*_ = *P*_*f*_ − π_*f*_, *P*_*f*_ and π_*f*_ are the turgor and osmotic fruit pressures, and *T* is transpiration. The turgor pressure of the fruit is estimated by solving the Lockhart's equation (Lockhart, 1965).

### Experimental data

In order to obtain input data for the parameterization and calibration of QualiTree, a study was carried out during 2011 on a commercial peach orchard in Golmekan, Iran (36° 29′ N, 59° 17′ E, 1,176 m above sea level). The soil is sandy loam (64% sand, 30% silt) with pH 7.51 and 2.5 m deep. The average annual rainfall in the study area is 212 mm. Rainfall, temperature, and reference evapotranspiration were recorded at a weather station located close to the orchard.

The experiment concerned a mid-late maturing cultivar of *P. persica* L. (cv. “Elberta”) planted in 2003. Trees were grafted on G.H. Hale rootstock and spaced 4 × 5 m apart. Trees were managed according to the usual practices in the region, including fertilization, weed, and pest control. Thinning was performed before the establishment of the irrigation treatments and led to a crop load of, approximately, 280 fruits per tree.

Irrigation treatments were arranged in a randomized complete block design with four replications. Water was applied through a drip irrigation system with two lateral pipes per row and 6 emitters (different flow discharge for each treatment) per tree. Three different irrigation treatments were considered in the current study: (i) a low stress (LS), applying an equivalent of 7.2 mm day^−1^ of water; (ii) a moderate stress (MS), applying 3.6 mm day^−1^; and (iii) a severe stress (SS), where 1.8 mm day^−1^ of water were applied to the trees. These treatments were imposed from the mid-pit hardening stage (12 June) until harvest (23 September). After the treatment onset, the rainfall was very limited with only 5 mm in July, 1 mm in August and September. The PET (Potential EvapoTranspiration) ranged from 5 up to 17 mm day^−1^ (mean value ~11 mm day^−1^). Irrigation was managed with a constant duration per day, 5 days per week, and with no interaction with PET calculation. Indeed, water amount by irrigation only partly compensated PET. As the irrigation was, even for the low stress limited to 7.2 mm day^−1^, water deficit increased during the season.

Further details on the experimental setup and water amounts applied can be found in Rahmati et al. (2015a).

The measurements (tree water status, gas exchange, growth, and fruit composition) were undertaken on the central trees (one per replication, thus four per treatment) to avoid border effect.

Tree water status was assessed through leaf (Ψ_*leaf*_) and stem (Ψ_*stem*_) water potential measurements performed on a weekly basis. Determinations of Ψ_*leaf*_ were carried out at predawn and midday, whereas those of Ψ_*stem*_ were performed at midday. Measurements were performed on 3 fully-expanded mature leaves per tree (exposed to direct solar radiation in the case of Ψ_*leaf*_) using a pressure chamber (ELE, UK). For Ψ_*stem*_ determinations, leaves were enclosed in plastic bags covered with aluminum foil for at least 2 h prior to measurements. Therefore, on each date, Ψ_*leaf*_ and Ψ_*stem*_ were measured on 12 leaves per treatment.

Under clear-sky conditions, leaf assimilation and transpiration were determined between 10 a.m. and solar noon using a portable gas exchange system (LCA-4, ADC, Hoddeson, England). Stomatal conductance (*g*_*s*_) was measured between 10 a.m. and solar noon using a leaf porometer (SC-1, Decagon Devices Inc., WA, USA). These measurements were performed on a fortnight basis, starting at the same date as tree water status determinations, and were taken on three fully-exposed upper-canopy leaves per tree.

Leafy shoot and fruit growth measurements were performed on 10 FU per tree (12% of the total number of FU). On each FU, the number of leafy shoots and fruits was recorded, and the length of leafy shoots and the cheek diameter of fruits were measured every week from bloom to harvest.

Over the growing season up to harvest (6 dates from 24 July to 23 September), three fruits per tree (12 per treatment) were sampled in order to determine fruit and stone fresh mass, dry mass (oven dried at 70°C) and dry matter content (Rahmati et al., 2015a,b). These fruits were also used for determining soluble sugar (sucrose, glucose, fructose, and sorbitol) contents using a high performance liquid chromatography protocol (Gomez et al., 2002).

Further details on field measurements and laboratory procedures, as well as the obtained data for the dynamics over the growing season of Ψ_*leaf*_, Ψ_*stem*_, and gas exchange, stomatal conductance, leafy shoot and fruit growth, and sugar contents have been published elsewhere (Rahmati et al., 2015a,b).

### Input data for QualiTree

Climate data (including global solar radiation, temperature, wind speed, and relative humidity) collected at a weather station located into the experimental field, were used as a model input. Water potential at the root collar was estimated using pre-dawn and minimal stem water potentials measured throughout the growing season for the 3 irrigation treatments applied to the experimental orchard (LS, MS, and SS). An additional virtual irrigation treatment was built, with values of water potential (predawn and minimal) corresponding to trees experiencing no water stress (NS), i.e., a predawn water potential around −0.3 MPa and midday water potential about −0.7 MPa over the entire season (Abrisqueta et al., 2015). In the case of LS, MS, and SS, the hourly water potential was extrapolated from experimental measurements, assuming a constant water potential during the night equal to the predawn water potential, and allowing for a sinus variation during the day from the predawn down to the minimal water potential and then up to the predawn water potential again. The water potential measurements were therefore used for each scenario of water stress.

Initial values for leafy shoot and fruit dry masses, and fruit sugar concentrations (sucrose, fructose, glucose, and sorbitol) were taken from the experimental data at the beginning of the season.

### Parameter estimation

Previous work (Mirás-Avalos et al., 2011) has shown that some parameters concerning the carbon economy in QualiTree are cultivar-dependent. Therefore, we parameterized the model for the “Elberta” cultivar. Experimental data were used to estimate most of the model parameters shown in Table 1. All other parameters were taken from published reports (i.e., Génard et al., 1998; Lescourret et al., 1998; Mirás-Avalos et al., 2011), as presented in Supplementary Table 3.

**Table 1:** Parameter values concerning carbon economy and sugar development in QualiTree estimated in the current study for cv. “Elberta” under Iranian semi-arid conditions.

**Parameter**	**Definition (corresponding equation)**	**Unit**	**Value**	**Range**
**SPECIFIC PARAMETERS FOR CHANGES OF STOMATAL CONDUCTANCE (g**_s_**) ACCORDING TO ENVIRONMENTAL AND PLANT CONDITIONS**				
*gs*_*max*_	Maximum g_s_	mol m^−2^ s^−1^	0.0796	–
*a*_*vpd*_	Jarvis parameter expressing the effect of VPD on g_s_	hPa^−1^	0.158	
*b*_*vpd*_	Jarvis parameter expressing the effect of VPD on g_s_	Dimensionless	8.06	
*a*_*temp*_	Jarvis parameter expressing the effect of temperature on g_s_	°C^−1^	0.23	
*b*_*temp*_	Jarvis parameter expressing the effect of temperature on g_s_	Dimensionless	22.5	
*a*_*psi*_	Jarvis parameter expressing the effect of PSI on g_s_	MPa^−1^	−8.467	
*b*_*psi*_	Jarvis parameter expressing the effect of PSI on g_s_	Dimensionless	13.129	
*a*_*par*_	Jarvis parameter expressing the effect of PAR on g_s_		0.005	
**GLOBAL PARAMETERS**				
Δ*psi*_*max*_	Maximum difference between stem and leaf water potential	MPa	0.447	–
**SPECIFIC PARAMETERS FOR LEAFY SHOOTS**				
*dd*_*min*_	Minimum degree-day value	Degree-day	0	0–600
*dd*_*max*_	Maximum degree day value	Degree-day	2,500	785–2,800
$RGR_{ls}^{ini}$	Leafy shoot initial relative growth rate	Degree-day^−1^	0.00036	0.001–0.01844
$DM_{ls}^{max}$	Potential dry mass of leafy shoot	g	4.65	6.5–66
*SLA*	Specific leaf area	m^2^ g^−1^	0.0158	0.014–0.016
**SPECIFIC PARAMETERS FOR WATERSPROUTS**				
*dd*_0_	Emergence of watersprouts	Degree-day	950	–
*dd*_*min*_	Minimum degree-day value	Degree-day	2,400	
*dd*_*max*_	Maximum degree day value	Degree-day	2,800	
$RGR_{ws}^{ini}$	Water sprout initial relative growth rate	Degree-day^−1^	0.0059	
$DM_{ws}^{max}$	Potential dry mass of watersprouts	g	11.1	
**SPECIFIC PARAMETERS FOR FRUITS**				
*dd*_*min*_	Minimum degree-day value	Degree-day	2,600	295.48–839
*dd*_*max*_	Maximum degree day value	Degree-day	2,800	986.93–3,500
$RGR_{f}^{ini}$	Fruit initial relative growth rate	Degree-day^−1^	0.0025	0.001439–0.0107
$DM_{f}^{max}$	Potential dry mass of fruits at maturity	g	35	32.5–59.22
*fleshDmc*	Flesh dry matter content	Dimensionless	0.195	0.09–0.16
*fs*_*sat*_	Saturation value for assimilate distribution between stone and flesh	g	3.87	5.8
*fs*_*slope*_	Initial slope for assimilate distribution between stone and flesh	g^−1^	0.2164	0.1
**PARTITIONING OF CARBON FLOW FROM THE PHLOEM INTO SUGARS**				
*k_1_*	Proportion of carbon as sucrose in the phloem sap	Dimensionless	0.3573	0.35–0.48
*K_3_*	Relative rate of sorbitol transformation to fructose	Day^−1^	0.0536	0.04–0.6
*K_5_*	Relative rate of sorbitol transformation to glucose	Day^−1^	0.0598	0.13–0.44
*K_2,1_*	Relative rate of decrease of k_2(t)_	Day^−1^	0.0879	0.05–0.13
*K_2,2_*	Time at which k_2(t)_ = 1 day^−1^	Day	74.0405	57–80
*K_4,1_*	Ratio of the relative rate of glucose and fructose transformation to the relative growth rate	Dimensionless	2.3380	2.2–4.5
**WATER STRESS EFFECTS ON VEGETATIVE GROWTH**				
*Ψ_*min*_*		MPa	−1.949	–
*Ψ_*max*_*		MPa	−1.378	
**WATER STRESS EFFECTS ON PHOTOSYNTHESIS**				
*A_*h*_*		MPa^−1^	0.3647	0.22
*B_*h*_*		MPa	6.3667	4.43

*Ranges of values for these parameters found in the literature are also shown. Ranges for the different parameters were taken from Harrison et al. (1989), Lescourret and Génard (2005), Mirás-Avalos et al. (2011, 2013a,b), and Wu et al. (2012)*.

Data from the water potential and gas exchange measurements were used for determining the parameters relative to the effects of water stress on photosynthesis and growth; as well as those referred to the Jarvis (1976) equation for simulating stomatal conductance. These parameters were estimated using non-linear least square regressions using the “nls” function in R software version 3.2.4 (R Core Team, 2016).

Potential fruit, leafy shoot, and water sprout growth parameters, namely the initial relative growth rate (*RGR*^*ini*^), maximal dry mass (*DM*^*max*^), and minimum and maximum degree-days (*dd*_*min*_, *dd*_*max*_) were estimated by non-linear least square regressions using the highest data issued from the field experiment (90% quantile).

The parameters for the sugar sub-model were estimated by a Nelder-Mead optimization algorithm for derivative-free optimization (“nmkb” function in R version 3.2.4, R Core Team, 2016).

Two parameters were estimated globally by running QualiTree: the parameters expressing the effect of water deficit on tree growth limitation (Ψ_*min*_ and Ψ_*max*_, Equation 7). The criterion (A) to be minimized for this estimation (using the “optim” function in R version 3.2.4; R Core Team, 2016) was a weighted sum of differences averaged over the FU for leafy shoot, fruit, and water sprout dry masses:

(9) $$\begin{array}{l} A=\frac{1}{\sigma_{x}^{2}}\frac{1}{n}\sum_{i}\left[\frac{1}{n_{i}}\sum_{j=1}^{n_{i}}{\left(x_{ij}-x_{ij}^{s}\right)}^{2}\right]\\ \;+\frac{1}{\sigma_{y}^{2}}\frac{1}{n}\sum_{i}\left[\frac{1}{n_{i}}\sum_{j=1}^{n_{i}}{\left(y_{ij}-y_{ij}^{s}\right)}^{2}\right]\\ \;+\frac{1}{\sigma_{z}^{2}}\frac{1}{n}\sum_{i}\left[\frac{1}{n_{i}}\sum_{j=1}^{n_{i}}{\left(z_{ij}-z_{ij}^{s}\right)}^{2}\right] \end{array}$$

where *x*_*ij*_*, y*_*ij*_, and *z*_*ij*_ are the observed averages for leafy shoot, water sprout, and fruit dry mass per FU (i) and per date (j), respectively; $x_{ij}^{s}$, $y_{ij,}^{s}$ and $z_{ij}^{s}$ the corresponding simulated average values of leafy shoot, water sprout and fruit dry mass per FU (i) and date (j), respectively; *n*_*i*_, the number of dates for each FU (i); *n*, the total number of FU; $\sigma_{x}^{2}$, the variance of *x*_*ij*_; $\sigma_{y}^{2}$, the variance of *y*_*ij*_; and $\sigma_{z}^{2}$, the variance of *z*_*ij*_.

### Comparison of observed and simulated values

The Relative Root Mean Square Error (RRMSE) was used to compare the mean observed and simulated values of leafy shoot, water sprout, and fruit dry masses as well as for sugar concentrations in fruit flesh. This index represents the mean distance between simulations and measurements, and is a criterion commonly used for assessing the goodness-of-fit of non-linear models (Kobayashi and Salam, 2000). It is defined as:

(10) $$RRMSE=\;\frac{1}{\bar{y}}\sqrt{\frac{1}{N}\sum_{i=1}^{N}{\left(y_{i}-y_{i}^{s}\right)}^{2}}$$

where *i* refers to a given FU on a given date, *y*_*i*_ is the observed value, *y*_*si*_ is the corresponding simulated value, *N* is the number of observed data, and ȳ is the mean of observed values. The smaller the RRMSE is, the most accurate the simulation is.

### Assessing the relative importance of different physiological processes at the tree scale

A virtual exercise using QualiTree for assessing the relative importance of photosynthesis and growth limiting functions on tree vegetative and reproductive development, as well as on fruit composition, was carried out. In order to do this, four different hypotheses were tested: (i) water deficit was assumed to have no effect on photosynthesis and growth, (ii) water deficit was assumed to limit only light-saturated leaf photosynthesis rate (acting on Equation 4, while Equation 7 was set to 1); (iii) water stress was assumed to limit vegetative growth only through turgor pressure reduction (acting on Equation 7, while Equation 4 was set to *P*_*max*0_); and (iv) water deficit was assumed to reduce both light-saturated leaf photosynthesis rate and vegetative growth (acting on both Equations 4 and 7). These scenarios were combined with those of irrigation (water stress). Finally, we compared the results of these simulations at harvest to a control scenario consisting of no water stress conditions, thus inducing no limitations for growth and photosynthesis. On the whole, 13 scenarios were analyzed.

The output variables were the growth in dry mass of the different tree compartments (roots, wood, leafy shoots, water sprouts, and fruits), fruit fresh mass, C assimilation at the whole-tree level, and the concentrations of sucrose, glucose, fructose, and sorbitol in the fruit flesh.

## Results

### Parameterization of the model and simulation of plant water potential

**Figure 2:**
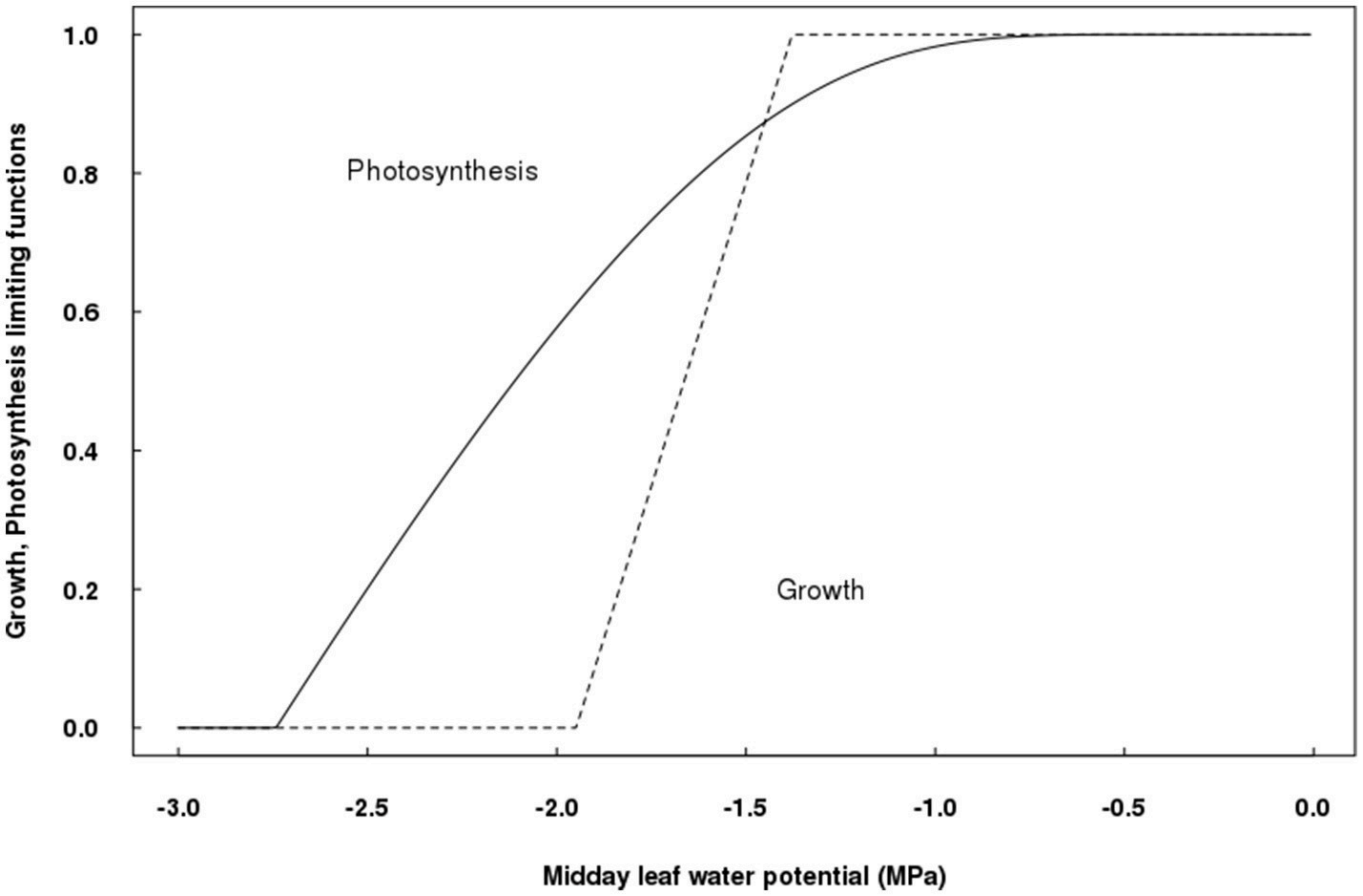
Differential sensitivity of potential vegetative growth- and photosynthesis-limiting functions to leaf water potential in QualiTree.

The parameters relating the effect of water stress on photosynthesis have been estimated, as well as those for the influence of water restrictions on vegetative growth (Table 1), allowing to plot photosynthesis and growth limitations as a function of Ψ_*leaf*_ (Figure 2). Photosynthesis started to decline at, approximately, −0.8 MPa, describing an exponential curve according to Equation (4); while vegetative growth started to decrease at about −1.4 MPa, although its declining rate was faster than that of photosynthesis, as described in Equations (5–7). Accordingly, growth was null for Ψ_*leaf*_ values lower than −1.94 MPa, whereas photosynthesis was still 60% of its maximum at this level of water deficit. Null photosynthesis occurred at Ψ_*leaf*_ values of −2.7 MPa (Figure 2).

**Figure 3:**
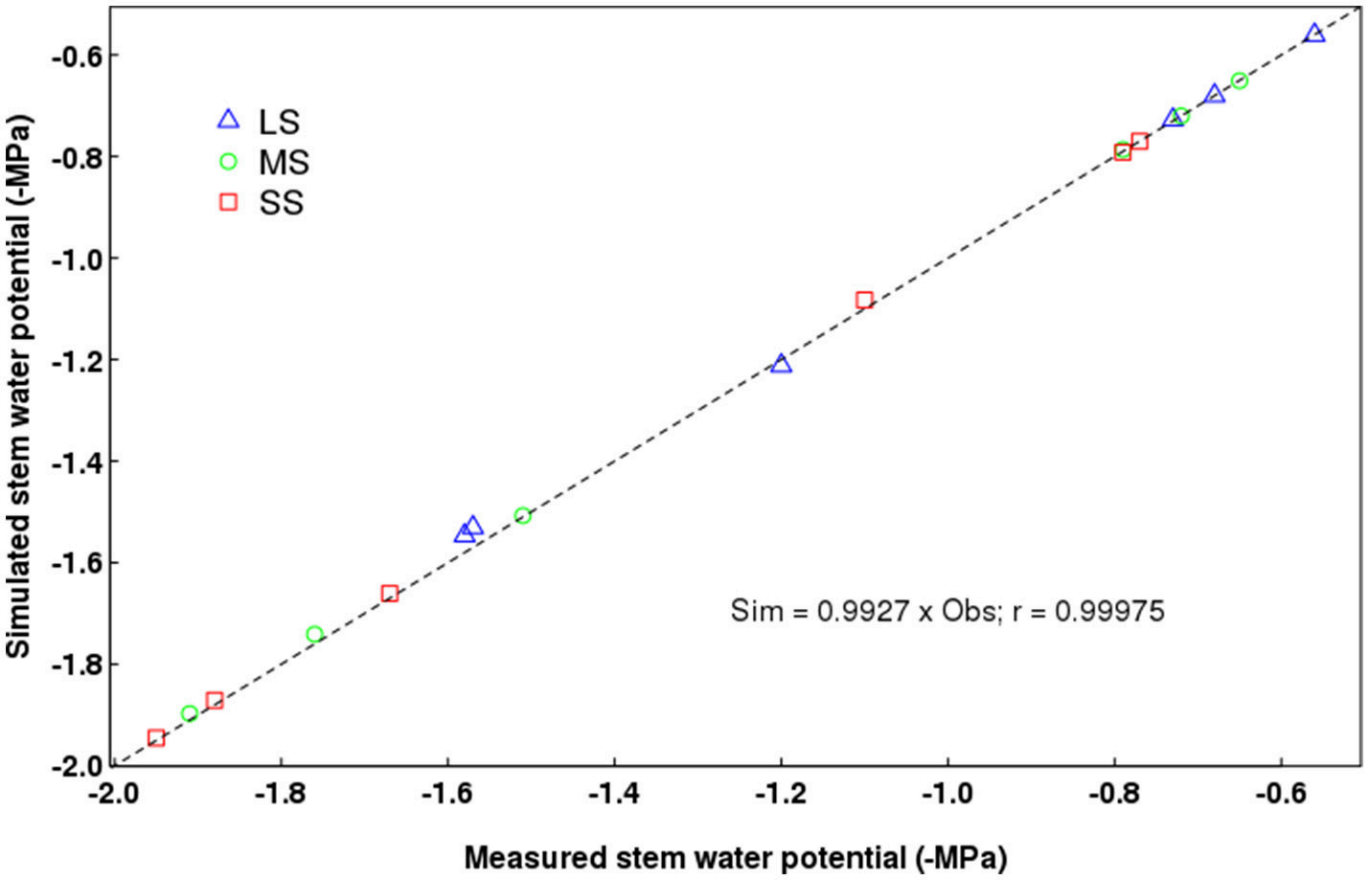
Relationship between measured and simulated stem water potentials. LS, low stress; MS, moderate stress; SS, severe stress. The dotted diagonal line denotes the 1:1 ratio.

**Figure 4:**
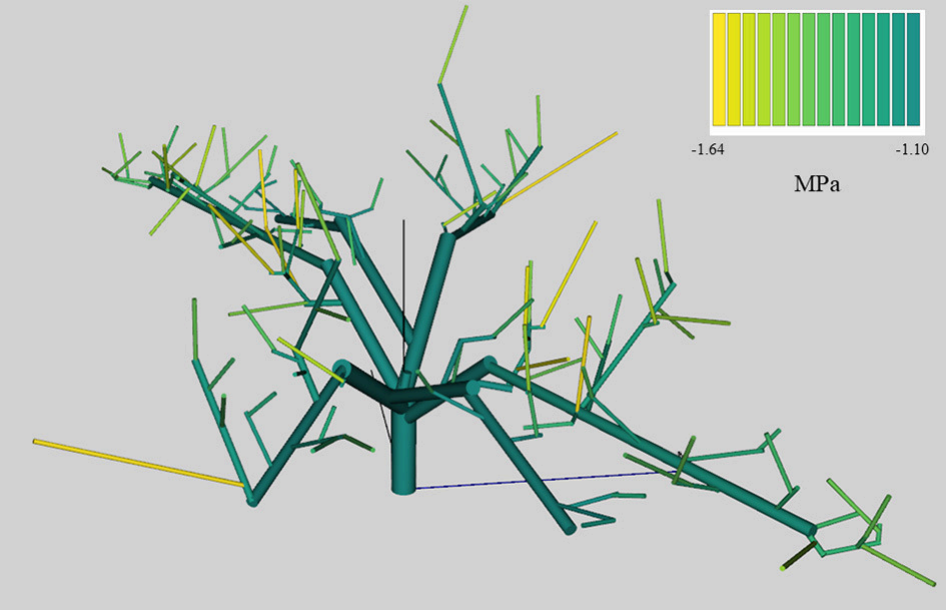
Stem water potential at 14 h in mid-July (14th) of an Elberta peach tree, under the low stress (LS) treatment, simulated by QualiTree. The colors represent the stem water potential values (MPa) for each FU.

On average, QualiTree was able to correctly simulate the Ψ_*stem*_ measured values (Figure 3). A strong correlation between observed and simulated values was obtained. Figure 4 shows the variability of Ψ_*stem*_ values among FU for the tree under the LS treatment at the beginning of the afternoon on July 14th 2011. This variability plays an essential role on the carbon acquisition and organ growth within the tree canopy, as well as on the differential development of fruit quality. As shown in Figure 4, the difference in Ψ_*stem*_ among FU can be as high as −0.5 MPa for the same date and hour; therefore, on the same tree, some FU are exposed to low water stress, whereas others were suffering from moderate to severe water stress. Supplementary Figures 1, 2 display, for the same date and hour as Figure 4, the between-FU variability of photosynthesis by surface unit and incident PAR for each FU, respectively. Those FU that were less water-stressed (Figure 4) had a higher photosynthetic capacity (Supplementary Figure 1) than those that were more water-stressed.

### Model fitting: fruit, leafy shoot, and water sprout dry masses

**Figure 5:**
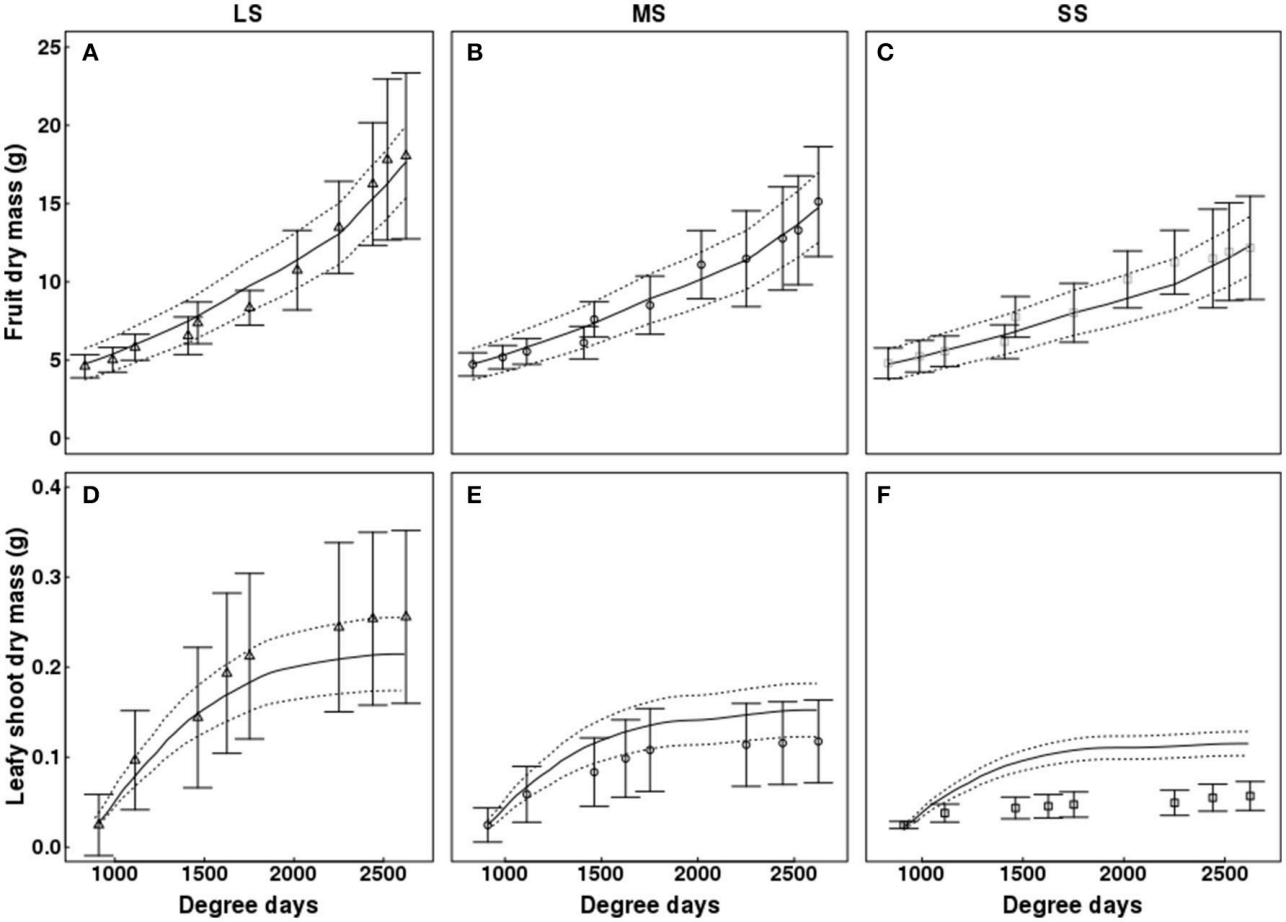
Test of the model against experimental data for the Elberta trees grown under different irrigation levels in the semi-arid conditions of Iran. Variation of fruit **(A–C)** and leafy shoot growth **(D–F)** among monitored shoots as a function of degree-days, either observed (points) or simulated (solid lines). Vertical bars and dotted lines indicate standard deviation for observed and simulated values, respectively. Harvest time was 2,625 degree-days. Results from the LS tree corresponded to the calibration, whereas those from MS and SS trees refer to the validation of the model. LS, low stress; MS, moderate stress; SS, severe stress.

**Figure 6:**
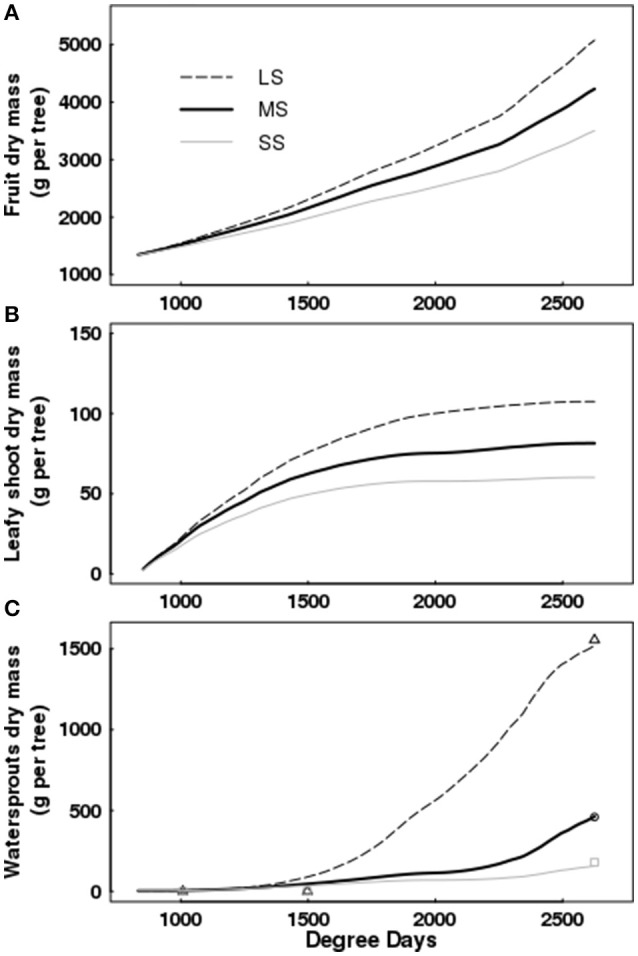
Simulated cumulative dry mass of fruits **(A)**, leafy shoots **(B)**, and water sprouts **(C)** as a function of degree days for Elberta trees under different irrigation levels. Dynamic changes in water sprout dry mass, either observed (points) or simulated (lines). Harvest time was 2,625 degree-days. LS, low stress; MS, moderate stress; SS, severe stress.

QualiTree reproduced correctly the patterns of growth in dry mass for leafy shoots, fruits, and water sprouts for the different water availability scenarios (Figures 5, 6). In the case of fruits, simulated values fitted correctly those observed for the three irrigation treatments (Figure 5). RRMSE values were 0.08, 0.06, and 0.09 for LS, MS, and SS, respectively. Simulated leafy shoot dry masses fitted correctly the observed values for LS, although with a slight underestimation, whereas QualiTree overestimated the mean values for MS and SS (Figure 5). RRMSE values were 0.17, 0.65, and 2.40 for LS, MS, and SS, respectively. Despite these results, QualiTree reproduced the decreased variability observed in leafy shoot growth with increasing water stress intensity, as pointed out by the standard deviation in the simulated values (Figure 5). The total dry mass of water sprouts, which represented 35% of the total leaf area at harvest on average, was correctly simulated by QualiTree for the three irrigation treatments (Figure 6). RRMSE values were 0.11, 0.22, and 0.21 for LS, MS, and SS, respectively.

Simulations showed that increasing water stress intensity had a more pronounced effect on water sprouts and on leafy shoots than on fruit growth (Figure 6). The dry mass of water sprouts diminished by 70 and 91% for MS and SS with respect to the control, whereas leafy shoot dry mass was reduced by 29 and 44% under MS and SS; finally, fruit dry mass decreased by 17 and 31% under MS and SS treatments.

### Model fitting: fruit fresh mass and sugar concentrations

**Figure 7:**
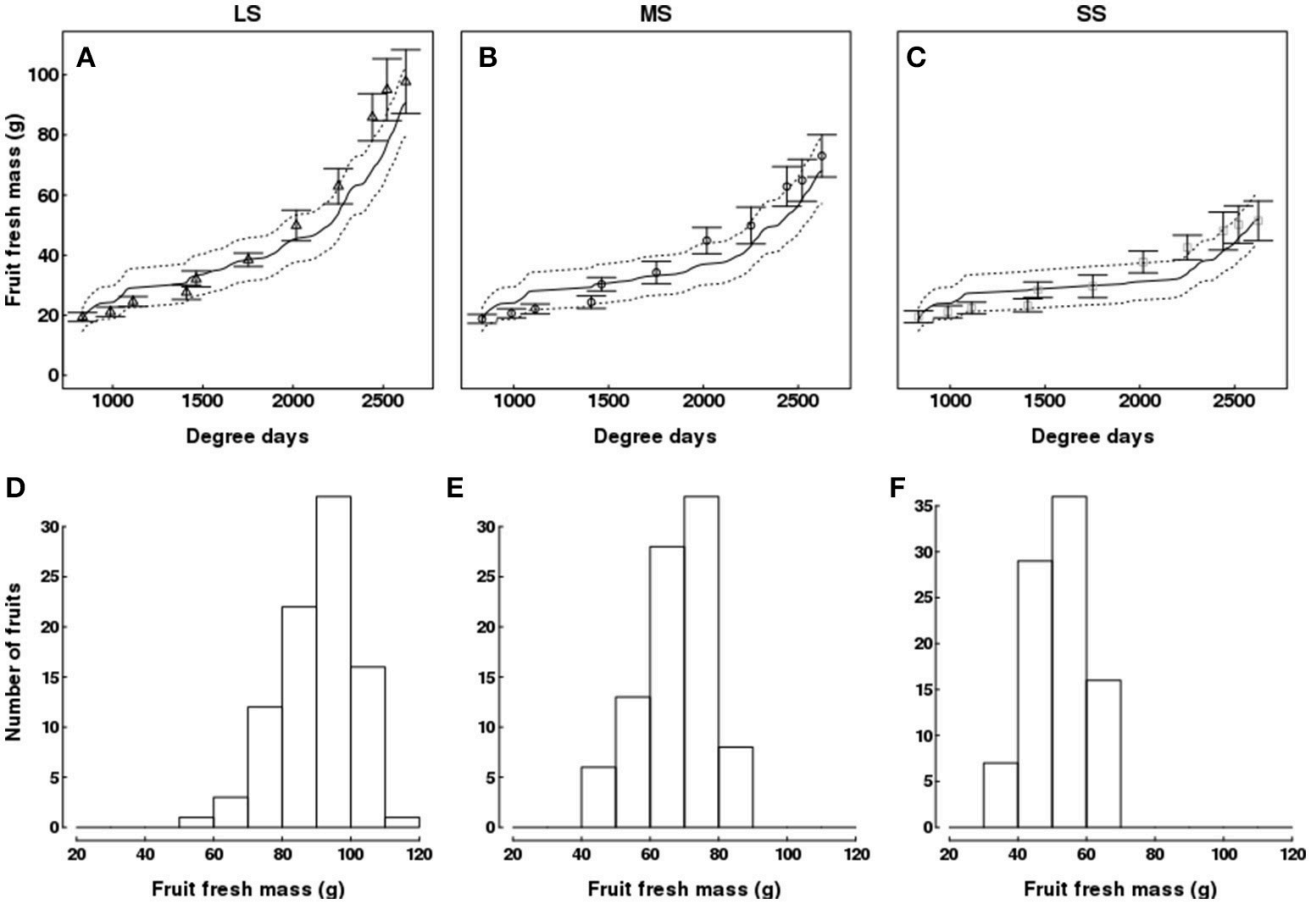
Test of the model against experimental data for the Elberta trees grown under different irrigation levels in the semi-arid conditions of Iran. Variation of fruit fresh mass **(A–C)** among monitored shoots as a function of degree-days, either observed (points) or simulated (solid lines). Vertical bars and dotted lines indicate standard deviation for observed and simulated values, respectively. Harvest time was 2,625 degree-days. Simulated fruit fresh mass distributions at harvest for the LS **(D)**, MS **(E)**, and SS **(F)** treatments. LS, low stress, MS, moderate stress; SS, severe stress.

The model slightly underestimated fruit fresh mass near harvest (Figures 7A–C). QualiTree showed that fruit size class distribution at harvest was shifted toward smaller sizes as water deficit intensified (Figures 7D–F). Moreover, the dry matter content increased with the intensity of water stress (Supplementary Figure 3).

**Figure 8:**
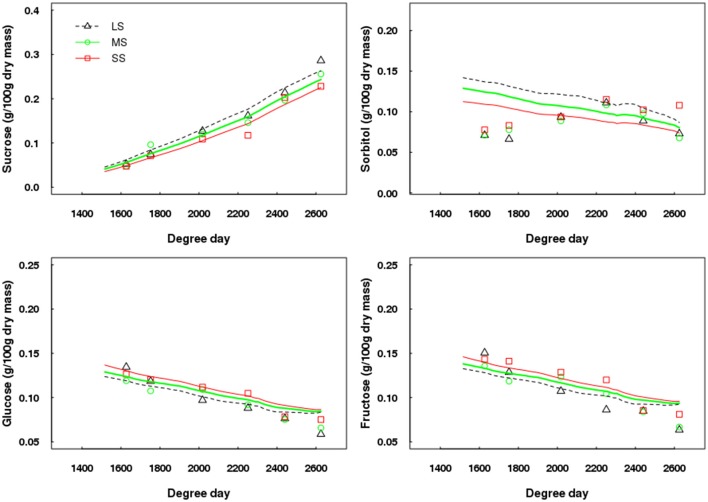
Test of the model against experimental data for the Elberta trees grown under different irrigation levels in the semi-arid conditions of Iran. Variations of sugar contents on a dry mass basis among monitored shoots as a function of degree-days, either observed (points) or simulated (lines). Harvest time was 2,625 degree-days. Results from the LS tree corresponded to the calibration, whereas those from MS and SS trees refer to the model validation. LS, low stress, MS, moderate stress; SS, severe stress.

Concerning fruit composition, the simulated patterns of sucrose, glucose, and fructose contents in the fruit flesh fitted well the experimental data (Figure 8). In contrast, sorbitol was not correctly simulated at the beginning of its development within the fruit (Figure 8), but the simulated values were very similar to those measured at harvest. The general trends were correctly simulated, namely glucose and fructose contents tended to be higher and sucrose content to be lower with increased water deficit intensity. However, in the case of sorbitol, the simulated trends were not in accordance with measured data. The RRMSE values for sucrose, glucose, fructose and sorbitol ranged between 0.09–0.10, 0.08–0.15, 0.09–0.17, and 0.26–0.49, respectively.

When considering concentrations of sugars on a fresh mass basis, the general trends over the season were correctly estimated by QualiTree, except for sorbitol, but the model tended to slightly overestimate the observed values (Supplementary Figure 4).

### Relative importance of the reduction in source activity and potential vegetative growth on QualiTree outputs

Irrigation level exerted a significant influence on all the outputs except for leafy shoot dry mass (Supplementary Table 4).

**Figure 9:**
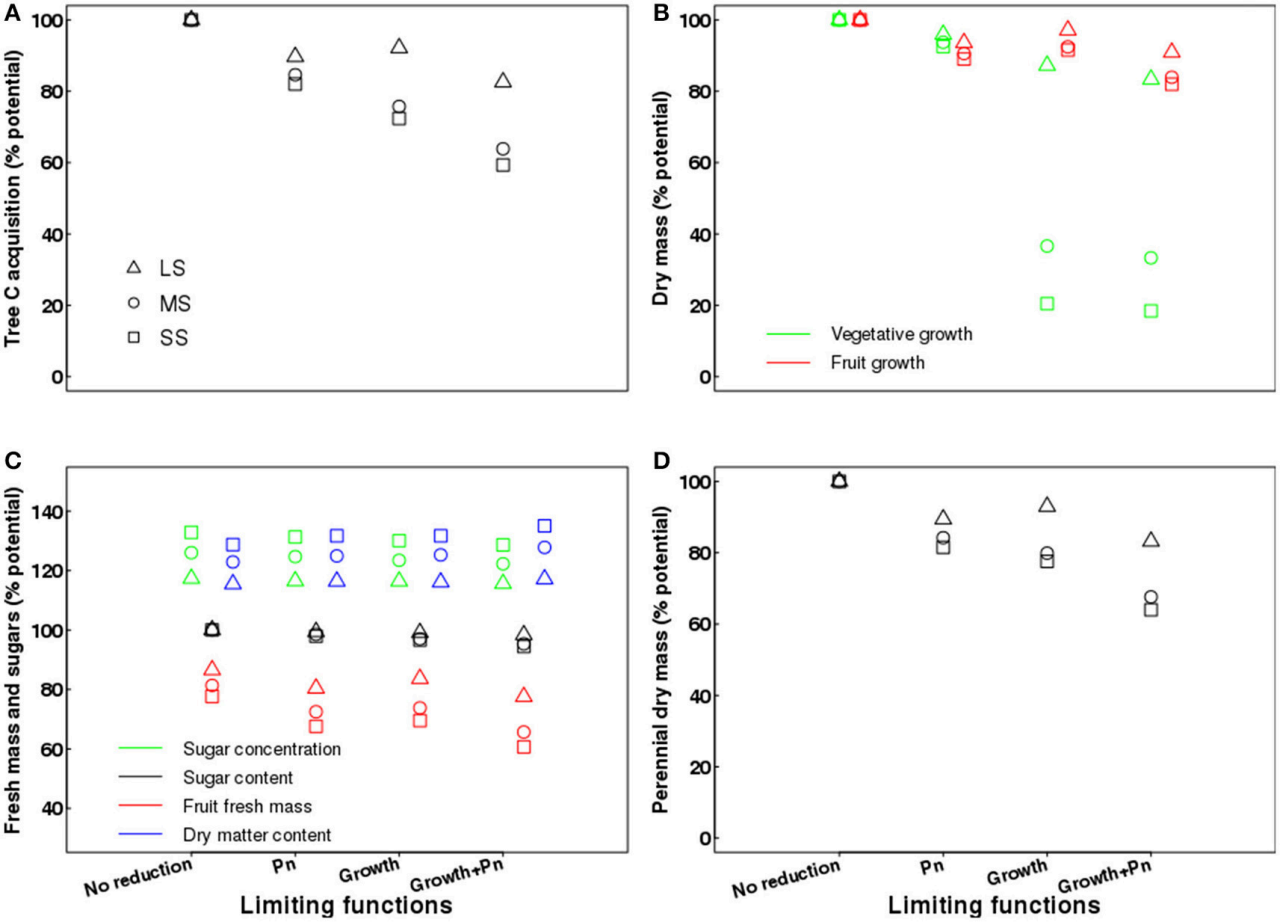
Effects of limiting functions (photosynthesis and growth) on tree carbon acquisition over the growing season **(A)**; vegetative (leafy shoots, water sprouts and roots younger than 1 year) and fruit growth in dry mass over the growing season **(B)**; fruit fresh mass, sugar concentration, sugar content and dry matter content of the fruits **(C)**; and growth in dry mass of perennial organs (trunk, branches, and roots older than 1 year) over the growing season **(D)** under different irrigation levels, expressed as percentage of the value obtained in a well-watered control (no limiting functions). LS, low stress; MS, moderate stress; SS, severe stress; *Pn*, photosynthesis.

In the case of the virtual control named “No reduction,” meaning that water deficit did not impact photosynthesis and vegetative growth, the fruit was the only organ affected, with growth and composition altered (Figure 9). In this case, the fruit growth in fresh mass was reduced due to a limitation of the water import in the fruit, but the dry matter content and the fruit sugar concentrations increased due to a lesser dilution effect (Figure 9C).

In the case where only limitations on light-saturated photosynthesis rate due to water stress were assumed, tree carbon acquisition was reduced by 10, 15, and 18% for the LS, MS, and SS treatments, respectively (Figure 9A), when compared to a well-watered control where photosynthesis was always maximal. Vegetative growth in dry mass (leafy shoots + water sprouts + new roots) was slightly affected by water stress, with reductions up to 7.5% for the SS treatment (Figure 9B). Fruit dry mass decreased by 6, 9, and 11% for LS, MS, and SS treatments, respectively. In contrast, fruit fresh mass was severely restricted not only by photosynthesis limitations linked to water deficit, but also for the direct effect of water stress on water flow to fruit, with reductions of 20, 27, and 32% for LS, MS, and SS, respectively. This led to increasing fruit dry matter content (up to 34% in SS) and also to greater concentrations of sugars, from 19% in LS to 31% in SS (Figure 9C). However, fruit sugar content was almost unaffected by water stress, with reductions from 0.6% in LS to 2% in SS (Figure 9C). Finally, the growth in dry mass of the perennial organs (trunk, branches, old roots, and stem wood) declined by 10% in LS and 19% in SS (Figure 9D).

When assuming exclusively a direct effect of tree water status on potential vegetative growth, tree C acquisition was reduced up to 28% for SS when compared to a well-watered control (Figure 9A). Water restrictions also led to severe declines in vegetative growth in dry mass of 13, 63, and 79% for LS, MS, and SS, respectively (Figure 9B). Fruit dry mass was slightly reduced by water stress, up to 8% in SS, whereas fruit fresh mass declined significantly with increasing water deficit, from 16% in LS to 30% in SS (Figure 9C), due to the combined effect of water stress limitations on fruit growth and water inflow. In contrast, fruit dry matter content and the sugar concentrations in the fruit increased up to 32% under SS (Figure 9C). However, fruit sugar content was almost unaffected by water stress, with reductions from 1% in LS to 3.3% in SS (Figure 9C). Finally, the growth in dry mass of perennial organs was reduced by 7% in LS and 22% in SS (Figure 9D).

The fourth set of scenarios assumed that water restrictions affected both photosynthesis and growth; however, the individual effects of each limiting function were not strictly additive. With respect to a well-watered control, tree C acquisition decreased by 17, 36, and 41% for LS, MS, and SS treatments, respectively (Figure 9A). Vegetative growth in dry mass declined by 17% in LS and 82% in SS, whereas fruit dry mass was less affected and only decreased by 18% under SS conditions (Figure 9B). Fruit fresh mass declined by 22% in LS and 39% in SS, whereas the fruit dry matter content and sugar concentrations increased approximately by 30% in SS (Figure 9C). However, fruit sugar content was almost unaffected by water stress, with reductions from 1.6% in LS to 5.5% in SS (Figure 9C). Finally, the growth in dry mass of perennial organs decreased by 17, 32, and 36% in the LS, MS, and SS treatments, respectively (Figure 9D).

## Discussion

### Improvements in QualiTree, parameterization, and validation

A late-maturing peach cultivar (“Elberta”), grown under semi-arid conditions, was successfully implemented and parameterized into QualiTree. The newly estimated parameters of leafy shoot and fruit growth, as well as those for fruit composition, in this cultivar were within previously reported ranges (Lescourret and Génard, 2005; Mirás-Avalos et al., 2011, 2013a,b; Wu et al., 2012).

The implementation of the energy balance and water transfer modules into QualiTree allowed for simulating the light-interception for the NIR and TIR wavebands, apart from direct and diffuse PAR, similarly to a previous model for tomato (Baldazzi et al., 2013). With these improvements, the effects of water restrictions on photosynthesis and growth can be separately estimated for each FU.

As a novelty, a direct effect of water stress on vegetative growth has been included in QualiTree. The parameters of this limiting function showed that vegetative growth would be null when a Ψ_*leaf*_ threshold of −1.94 MPa is reached. This value is in the order of magnitude of water potentials that greatly reduced vegetative growth in mid-late maturing peach cultivars: −1.5 MPa (Ψ_*stem*_) for “Andross” (Girona et al., 2005) and −1.8 MPa (Ψ_*stem*_) for “Catherine” (Mirás-Avalos et al., 2016). In contrast, light-saturated photosynthesis rate would be zero at more negative Ψ_*leaf*_ values (−2.7 MPa), in accordance with experimental evidence (Chaves et al., 2002; Tardieu et al., 2011). Therefore, QualiTree was able to account for the different sensitivities of growth and photosynthesis to water stress, leading to an uncoupling of both processes under water deficit conditions (Muller et al., 2011).

These improvements within the model allowed for simulating large variations in carbon and water availability within the tree. QualiTree simulations fitted well the general patterns of fruit growth and sugar development observed in the experimental data. In addition, simulated fruit size distributions at harvest tended to shift to smaller fruit sizes with increasing water stress, in agreement with field experiments (Bryla et al., 2005). Moreover, fruit fresh weight variability was reduced with increasing water deficit intensity, in accordance with experimental data. Fruits are the strongest carbohydrate sinks on the tree during the last half of the period of fruit development. However, our simulations showed that fruit growth in dry mass was much less sensitive to water restrictions than fruit fresh mass, as reported in several field studies (Berman and DeJong, 1996; Girona et al., 2006). On the whole, QualiTree was able to reproduce reasonably well the behavior of peach vegetative and fruit growth under water restrictions, even though vegetative growth was overestimated.

In the current work, water sprouts were included for the first time in QualiTree by using an equation similar to that describing leafy-shoot potential growth. This allowed for a reasonable estimation of water sprout growth in dry mass and sets a step forward for understanding the physiological functions of these organs, which are not yet elucidated (Bussi et al., 2011). Water deficits have a huge effect on water sprout growth. Indeed, under SS, the water sprout mass was only 10% that of the LS treatment, indicating that the growth of this compartment is the most sensitive to water availability. From the practical perspective, water sprout emergence and further growth are not desirable for growers; therefore, water sprouts are often pruned in the orchard, and such practice is time-consuming and costly. Hence, water management could be a useful tool to control tree vigor and, especially, water sprout occurrence.

Apart from fruit growth and its within-tree variability, QualiTree correctly simulated the seasonal patterns of sucrose, glucose and fructose contents in the fruit flesh under different irrigation schedules. According to experimental research (Lo Bianco et al., 2000; Thakur and Singh, 2012), higher glucose and fructose, and lower sucrose contents are expected as a result of increasing water deficit; this pattern was well reflected by QualiTree. However, the model predicted lower sorbitol contents for the most water-stressed trees, which disagrees with current knowledge (Lo Bianco et al., 2000; Thakur and Singh, 2012; Rahmati et al., 2015b), thus further improvements are necessary.

### Relative importance of the water stress reduction in source activity and sink strength at the whole-tree level on QualiTree outputs

QualiTree successfully captured the water deficit direct limitation of plant growth (Solari and DeJong, 2006; Tardieu, 2013) and its secondary reduction of leaf photosynthesis and thereby carbon allocation to sink organs (Chaves et al., 2002), allowing for correct simulations of the effects of water deficit on tree growth, fruit size, and composition. Model outputs matched previous knowledge about the different sensitivities to water stress of vegetative and fruit growth in dry mass (Berman and DeJong, 1996). QualiTree predicted null vegetative growth when Ψ_*leaf*_ was more negative than a threshold of −1.94 MPa, while photosynthesis was still acting at 60% of its potential rate, consistently with the resilience to water deficit of this latter process (Chaves et al., 2002). Null photosynthesis was predicted at a Ψ_*leaf*_ threshold of −2.7 MPa, slightly more positive than the −3.5 MPa observed for prunes (Lampinen et al., 2004). These results agree with the fact that carbon demand (growth) always decays before carbon supply (photosynthesis), a common feature in all plant species (as reviewed by Muller et al., 2011).

QualiTree outputs showed that the influence of water stress on the growth function exerted a greater effect on tree carbon acquisition and vegetative growth in dry mass than the limitation of the light-saturated photosynthesis rate caused by water deficit. Additionally, the model showed that fruit growth in dry mass was less sensitive to water shortage than vegetative growth, whatever reduction function was considered in the simulations, in accordance with experimental evidence for many plant species (Hsiao, 1973; Berman and DeJong, 1996; Tardieu et al., 2011).

The alterations caused by water restrictions in the sink strength of plant organs modify the allocation pattern of assimilated carbon. Field observations suggest that assimilates are allocated to organs with stronger sink abilities when the plant is subject to water constrains (Yuan et al., 2009). In this sense, the greater sensitivity to water stress simulated for leafy shoot growth when compared to that of fruits could be explained by the higher transpiration rate of leaves, which induced low leaf water potentials and then lower sink strength than fruits (Lescourret et al., 1998; Egea et al., 2012).

In contrast, the declining in fruit fresh mass and the increase in sugar concentrations due to water deficit were of the same order of magnitude when individually accounting for either photosynthesis or growth limitations. Interestingly, when combining both processes, the reduction observed in QualiTree outputs was not additive. Furthermore, it is worth noticing that the increasing of sugar concentrations in the fruit flesh induced by water stress was similar to the increase in dry matter content in the fruits, suggesting that the main process affected was the water inflow to the fruit (Génard et al., 2010) and not a larger sugar accumulation in the fruit. Dilution by water plays a relevant role in determining the concentration of soluble sugars (Génard et al., 2014; Dai et al., 2016) and it is known to be largely affected by environmental conditions and management practices (Kobashi et al., 2000).

## Conclusions

In the current study, QualiTree was used for assessing the effects that water stress may have on tree vegetative growth, fruit size, and composition after being parameterized and calibrated for a late-maturing peach cultivar. Interestingly, simulations reflected a greater importance of the direct effect of water restrictions on vegetative growth when compared to the effect on photosynthesis. Moreover, QualiTree was able to reproduce the observed effects of water stress on fruit mass and sugar contents, despite its simple formalisms, indicating that this modeling approach was adequate to simulate carbon allocation at the tree organ level under various water deficit conditions.

This study can provide useful information and background for plant modelers, horticulturists, and plant biologists. For plant modelers, QualiTree suggests the need to account for the effects of water restrictions on vegetative and fruit growth, photosynthesis, and stomatal conductance. For plant biologists, QualiTree provides a suitable framework for formulating and testing hypotheses on the relations of carbon balance, plant growth, and water stress, which can be useful for simulating climate change scenarios and assess their effects on tree functioning. For horticulturists, the model could be a useful tool to simulate the effect of different regulated deficit irrigation strategies and their impacts on fruit yield and composition.

## Author contributions

MR, GD, MG, MB, MA, and GV: Conceived and designed the experiments; MR: Carried out the experiments; MR, JM-A, PV, FL, MG, and GV: Designed the simulation exercise and carried out the modeling approach; MR, JM-A, MG, FL, and GV: Analyzed and discussed the data and wrote the manuscript.

### Conflict of interest statement

The authors declare that the research was conducted in the absence of any commercial or financial relationships that could be construed as a potential conflict of interest. The handling Editor declared a shared affiliation, though no other collaboration, with one of the authors JM-A.
