# Supplementary material for: Disentangling the Effects of Water Stress on Carbon Acquisition, Vegetative Growth, and Fruit Quality of Peach Trees by Means of the QualiTree Model
Source: Front Plant Sci. 2018 Jan 24;9:3. doi: 10.3389/fpls.2018.00003 (PMC5788000; doi:10.3389/fpls.2018.00003)
Supplement: Supplementary file 1 [file DataSheet1.doc]

Supplementary Material

# Disentangling the Effects of Water Stress on Carbon Acquisition, Vegetative Growth and Fruit Quality of Peach Trees by Means of the QualiTree Model

**M. Rahmati, J.M. Mirás-Avalos, P. Valsesia, G.H. Davarynejad, M. Bannayan, M. Azizi, F. Lescourret, M. Génard, G. Vercambre***

**Correspondence:** Gilles Vercambre: gilles.vercambre@inra.fr

# Supplementary Tables

The supplementary material includes two tables describing the inputs and outputs from QualiTree and a table indicating the values for all the parameters used in the simulations described in the current manuscript. Moreover, a table displaying the results from an ANOVA applied to the simulated results at harvest is included. We provide additional references for stating the origin of each parameter value used.

**QualiTree: inputs and outputs**

The tree system must be initialised in order to run QualiTree. Moreover, the numbers of leafy shoots and fruits for each fruiting unit (FU) are required, as are the tree architecture characteristics (diameter and lengths of trunk, scaffolds, branches and FU at full bloom) necessary to calculate the distances among tree entities exchanging carbon. Hourly climatic data series included solar radiation, temperature, relative humidity, wind and rainfall. Other inputs are leaf and stem water potentials that influence photosynthesis and water flow in the fruit, respectively (Table S1). Inputs can originate either from regular measurements or from models (Lescourret et al., 2011).

QualiTree is able to display and study the within-tree variability of fruit quality and of underlying variables (Lescourret et al., 2011) due to the great number of outputs it provides (Table S2). These outputs include the dry masses of each different tree entity and several fruit quality criteria, such as the concentrations of sucrose, glucose, fructose and sorbitol in the fruit. These variables react to different simulation scenarios (Mirás-Avalos et al., 2011).

**Table S1.** Inputs for QualiTree.

| Input | Unit | Scale | Process |
| --- | --- | --- | --- |
| *Climate* | | | |
| Latitude, longitude | rad | - | All processes |
| Altitude | m | - | All processes |
| Radiation | J cm^-2^ | Hourly | Photosynthesis |
| Relative air humidity | % | Hourly | Fruit transpiration |
| Mean temperature | ºC | Hourly | Maintenance and respiration costs |
| Minimum temperature | ºC | Hourly | Degree-days calculation |
| Maximum temperature | ºC | Hourly | Degree-days calculation |
| Rainfall | mm | Hourly | Energy balance |
| Wind | km | Hourly | Energy balance |
| *Water status* | | | |
| Collar water potential | MPa | Hourly | Water flow |
| *Light interception* | | | |
| Within-tree shadow coefficient | Dimensionless | Daily | Photosynthesis |
| Tree spacings | m | - | Global calculations |
| *Tree organ processes* | | | |
| Dimensions of the ellipsoids representing tree canopy | mm | - | All processes |
| Cutting planes | mm | - | All processes |
| Ellipsoid angle | rad | - | All processes |
| Diameters and lengths of each branch | mm | - | All processes |
| Insertion and phylotaxis angles | rad | - | All processes |
| Initial mass for each tree organ | g | - | All processes |
| Number of leafy shoots, fruits and water sprouts | - | - | All processes |
| Carbon reserves for each tree organ | % | - | Reserves |
| Degree-days | ºC day | Daily | All processes |

**Table S2.** Outputs from QualiTree.

| Output | Unit | Scale |
| --- | --- | --- |
| *Global tree* | | |
| Dry masses for old wood, coarse and fine roots | g | Hourly |
| Carbon reserves for old wood, coarse and fine roots | g C | Hourly |
| Respiration costs for old wood, coarse and fine roots | g C | Hourly |
| Carbon needs for growth of old wood, coarse and fine roots | g C | Hourly |
| ***Branches*** | | |
| Water flow | m^3^ s^-1^ | Hourly |
| Stem water potential | MPa | Hourly |
| Leaf water potential | MPa | Hourly |
| Leaf temperature | °C | Hourly |
| Photosynthesis per surface unit | gC h^-1^ m^-2^ | Hourly |
| Photosynthesis rate | gC h^-1^ | Hourly |
| Direct radiation (PAR) | μmol m^-2^ s^-1^ | Hourly |
| Diffuse radiation (PAR) | μmol m^-2^ s^-1^ | Hourly |
| Fraction of sunlit leaves | Dimensionless | Hourly |
| PAR received by sunlit leaves | μmol m^-2^ s^-1^ | Hourly |
| PAR received by shaded leaves | μmol m^-2^ s^-1^ | Hourly |
| *Leafy shoots and water sprouts* | | |
| Dry mass | g | Hourly |
| Carbon reserves | g C | Hourly |
| Respiration costs | g C | Hourly |
| Maximum photosynthesis rate | μmol CO_2_ m^-2^ s^-1^ | Hourly |
| Leaf area | m^2^ | Hourly |
| Carbon needs for growth | g C | Hourly |
| *Stem wood* | | |
| Dry mass | g | Hourly |
| Carbon reserves | g C | Hourly |
| Respiration costs | g C | Hourly |
| Carbon needs for growth | g C | Hourly |
| *Fruits* | | |
| Dry mass | g | Hourly |
| Fresh mass | g | Hourly |
| Flesh dry mass | g | Hourly |
| Fructose, glucose, sucrose and sorbitol concentrations in the fruit | g g fresh weight^-1^ | Hourly |
| Fructose, glucose, sucrose and sorbitol amounts in the fruit | g | Hourly |
| Carbon reserves | g C | Hourly |
| Respiration costs | g C | Hourly |
| Carbon needs for growth | g C | Hourly |
| Maximum photosynthesis rate | μmol CO_2_ m^-2^ s^-1^ | Hourly |

**QualiTree: parameters**

In this work, we identified new parameter values for a peach cultivar (‘Elberta’) grown under semi-arid conditions, as presented in the main text. In addition, some previously reported parameters were employed (Lescourret and Génard, 2005; Mirás-Avalos et al., 2011). In this supplementary material, the complete set of parameters used in the current study and their origins are given (Table S3).

**Table S3.** Parameter values concerning carbon economy (6 groups of parameters) and fruit quality in QualiTree for ‘Elberta’ peach cultivar grown under Iranian semi-arid conditions.

| Parameter | Definition |  | Unit | Value | Origin |
| --- | --- | --- | --- | --- | --- |
| ***Global parameters*** | | | | | |
| *SReq* | Shoot:root ratio at equilibrium |  | Dimensionless | 4.6 | Grossman and DeJong (1994a)  Rieger and Marra (1994)  Hipps et al. (1995)  Mediene et al. (2002) |
| *k* | Parameter expressing the effect of distance between organs on carbon exchange within the tree |  | Dimensionless | 0.006 | Mirás-Avalos et al. (2011) |
| *Δpsi_max_* | Maximum difference between stem and leaf water potential |  | MPa | 0.447 | This work |
| ***Water stress effects on vegetative growth*** | | | | | |
| *Ψ_min_* |  |  | MPa | -1.949 | This work |
| *Ψ_max_* |  |  | MPa | -1.378 | This work |
| ***Water stress effects on photosynthesis*** | | | | | |
| *A_h_* |  |  | MPa^-1^ | 0.3647 | This work |
| *B_h_* |  |  | MPa | 6.3667 | This work |
| ***Specific parameters for leafy shoots*** | | | | | |
| *R_1_* | Proportion of leaves in the structural dry weight of leafy shoots |  | Dimensionless | 0.70 | Ben Mimoun (1997) |
| *SLA* | Specific Leaf Area |  | m^2^g^-1^ | 0.0158 | This work |
| *P_1_* | Light-saturated maximal leaf photosynthesis |  | µmol CO_2_ m^-2^ s^-1^ | 20.14 | Ben Mimoun (1997) |
| *p_2_* | Specific parameter for photosynthesis calculation |  | µmol CO_2_ m^-2^ s^-1^ | 66.95 | Ben Mimoun (1997) |
| *R_1r_* | Proportion of leaf reserve carbon in the total reserve carbon of leafy shoots |  | Dimensionless | 0.70 | Ben Mimoun (1997) |
| *p_4_* | Specific parameter for photosynthesis calculation |  | µmol CO_2_ µmol photons^-1^ | 0.058 | Higgins et al. (1992) |
| *GRC_ls_* | Leafy shoot growth respiration coefficient |  | g g^-1^ | 0.10 | Penning de Vries et al. (1989) |
| *dd_min_* | Minimum degree-day value |  | degree-days | 0 | This work |
| *dd_max_* | Maximum degree-day value |  | degree-days | 2500 | This work |
| *RGR_ls_^ini^* | Leafy shoot initial relative growth rate |  | degree-days^-1^ | 0.00036 | This work |
| *DM_ls_^max^* | Leafy shoot maximal dry mass |  | g | 4.65 | This work |
| ***Specific parameters for fruits*** | | | | | |
| *dd_min_* | Minimum degree-day value |  | degree-days | 2600 | This work |
| *dd_max_* | Maximum degree-day value |  | degree-days | 2800 | This work |
| *RGR_f_^ini^* | Fruit initial relative growth rate |  | degree-days^-1^ | 0.0025 | This work |
| *DM_f_^max^* | Potential dry mass of fruits at maturity |  | g | 35 | This work |
| *P_f_* | Fruit photosynthetic rate |  | μmol CO_2_ g^-1^ s^-1^ | 7.22 x 10^-3^ | Lescourret et al. (1998) |
| *p5* | Parameter for calculation of photosynthesis |  | g^-1^ | 0.0311 | Lescourret et al. (1998) |
| *p6* | Parameter for calculation of photosynthesis |  | (m^2^ s)/μmol photon | 0.005 | Pavel and DeJong (1993) |
| *p7* | Parameter for calculation of photosynthesis |  | μmol photon/(m^2^ s) | 60 | Lescourret et al. (1998) |
| *p8* | Parameter for calculation of photosynthesis |  | (m^2^ s)/μmol photon | 0.01 | Lescourret et al. (1998) |
| *r3* |  |  | Dimensionless | 0.04 | Lescourret et al. (1998) |
| ***Parameters common to all organs*** | | | | | |
| *MRR_st_^b^* | 1-year-old stem and fine roots maintenance respiration rate at the reference temperature |  | gC gbiomass^-1^ s^-1^ | 9.93 x 10^-9^ | Grossman and DeJong (1994b) |
| *MRR_ow_^b^* | Trunk and coarse root maintenance respiration rate at the reference temperature |  | gC gbiomass^-1^ s^-1^ | 1 x 10^-9^ | Grossman and DeJong (1994b) |
| *MRR_ls_* | Leafy shoots maintenance respiration rate at the reference temperature |  | gC gbiomass^-1^ s^-1^ | 43.45 x 10^-9^ | Grossman and DeJong (1994b) |
| *MRR_f_* | Fruits maintenance respiration rate at the reference temperature |  | gC gbiomass^-1^ s^-1^ | 7.81 x 10^-9^ | DeJong and Goudriaan (1989) |
| *Q_10_^st^* | *Q*_10_ value for 1-year-old stem, old wood and fruits |  | Dimensionless | 1.96 | Grossman and DeJong (1994a) |
| *Q_10_^ls^* | *Q*_10_ value for leafy shoots |  | Dimensionless | 2.11 | Grossman and DeJong (1994a) |
| *Q_10_^cr^* | *Q*_10_ value for coarse and fine roots |  | Dimensionless | 2 | Grossman and DeJong (1994a) |
| *TetaRef* | Reference temperature |  | ºC | 20 | Grossman and DeJong (1994a) |
| *CC_ls_* | Carbon content for leafy shoots |  | g C g^-1^ | 0.4262 | Ben Mimoun (1997) |
| *CC_f_* | Carbon content for fruits |  | g C g^-1^ | 0.4242 | Ben Mimoun (1997) |
| *CC_ow_* | Carbon content for stem wood, old wood, coarse roots and fine roots |  | g C g^-1^ | 0.461 | Ben Mimoun (1997) |
| *r6_ow_* | Maximum ratio of reserves for old wood and roots |  | Dimensionless | 1 | Mirás-Avalos et al. (2013) |
| *r6_sw_* | Maximum ratio of reserves for stem wood |  | Dimensionless | 0.2 | Ben Mimoun (1997) |
| *r6_ls_* | Maximum ratio of reserves for leafy shoots |  | Dimensionless | 0.3 | Quilot et al. (2004) |
| *GRCow* | Growth respiration coefficient for old wood, coarse roots and stem wood |  | g C g^-1^ | 0.086 | Penning de Vries et al. (1989) |
| *GRCls* | Growth respiration coefficient for leafy shoots |  | g C g^-1^ | 0.1 | Penning de Vries et al. (1989) |
| *GRCf* | Growth respiration coefficient for fruits |  | g C g^-1^ | 0.0843 | DeJong and Goudriaan (1989) |
| *GRCnr* | Growth respiration coefficient for new roots |  | g C g^-1^ | 0.09 | Penning de Vries et al. (1989) |
| ***Reserve mobilization*** | | | | | |
| *Rm_ls_* | Leafy shoot and fine roots mobile fraction of reserves |  | Dimensionless | 0.026 | Lescourret and Génard (2005) |
| *Rm_ow_* | Old wood, coarse root and one year old stem wood mobile fraction of reserves |  | Dimensionless | 0.02 | Moing and Gaudillère (1992)  Ashworth et al. (1993)  Spann et al. (2008) |
| *CCR_ls_* | Carbon content in leafy shoot reserves |  | g g^-1^ | 42.62 x 10^-2^ | Ben Mimoun (1997) |
| *CCR_st_* | Carbon content in stem wood, trunk, coarse root and fine root reserves |  | g g^-1^ | 46.10 x 10^-2^ | Ben Mimoun (1997) |
| ***Growth for different structural parts*** | | | | | |
| *RGR_sw_^ini^* | Stem wood initial relative growth rate |  | degree-days^-1^ | 7 × 10^-4^ | Berman and DeJong (2003) |
| *RGR_ow_^ini^* | Old wood and coarse root initial relative growth rate |  | degree-days^-1^ | 9.5 × 10^-4^ | Mirás-Avalos et al. (2011) |
| ***Fruit quality parameters*** | | | | | |
| *λ_ph_* | Proportion of carbon as sucrose in the phloem sap |  | Dimensionless | 0.3573 | This work |
| *k_1,1_* | Relative rate of decrease of *k_1_* (*t*), the relative rate of sucrose transformation to glucose and fructose |  | day^-1^ | 0.0879 | This work |
| *k_1,2_* | Time at which *k_1_* (*t*) = 1 day^-1^ |  | day | 74.045 | This work |
| *k_2_* | Relative rate of sorbitol transformation to glucose |  | day^-1^ | 0.0598 | This work |
| *K_3_* | Relative rate of sorbitol transformation to fructose |  | day^-1^ | 0.0536 | This work |
| *K_4_* | Ratio of the relative rate of glucose and fructose transformation to the relative growth rate |  | Dimensionless | 2.338 | This work |
| *fleshDMC* | Flesh dry matter content |  | Dimensionless | 0.195 | This work |
| *fs_sat_* | Saturation value for assimilate distribution between stone and flesh |  | g | 3.87 | This work |
| *fs_slope_* | Initial slope for assimilate distribution between stone and flesh |  | g^-1^ | 0.2164 | This work |
| *c_su_* | Carbon content of sucrose |  | gC g^-1^ sucrose | 0.421 | Génard and Souty (1996) |
| *c_so_* | Carbon content of sorbitol |  | gC g^-1^ sorbitol | 0.395 | Génard and Souty (1996) |
| *c_g_* | Carbon content of glucose |  | gC g^-1^ glucose | 0.4 | Génard and Souty (1996) |
| *c_f_* | Carbon content of fructose |  | gC g^-1^ fructose | 0.4 | Génard and Souty (1996) |
| *a_x_* | Ratio of area of the composite membrane of the fruit area |  | Dimensionless | 0.0273 | Fishman and Génard (1998) |
| *D_w_* | Water density |  | g cm^-3^ | 1 | Fishman and Génard (1998) |
| *L_x_* | Conductivity of the composite membrane for water transport |  | g cm^-2^ bar^-1^ day^-1^ | 0.23328 | Fishman and Génard (1998) |
| *φ* | Cell wall extensibility coefficient |  | bar^-1^ day^-1^ | 0.24 | Fishman and Génard (1998) |
| *Y* | Threshold value of hydrostatic pressure needed for growth |  | bar | 5 | Fishman and Génard (1998) |
| *psat1* | Parameter for the calculation of the saturated vapour pressure |  | bar | 0.008048 | Fishman and Génard (1998) |
| *psat2* | Parameter for the calculation of the saturated vapour pressure |  | ºC^-1^ | 0.0547 | Fishman and Génard (1998) |
| *posmotre* | Parameter for the calculation of the osmotic pressure of the individual fruit |  | bar | 7.6 | Fishman and Génard (1998) |
| *Specific parameters for changes of stomatal conductance (g_s_) according to environmental and plant conditions* | | | | | |
| *g_smax_* | Maximum g_s_ |  | mol m^-2^ s^-1^ | 0.0796 | This work |
| *a_vpd_* | Jarvis parameter expressing the effect of VPD on g_s_ |  | hPa^-1^ | 0.158 | This work |
| *b_vpd_* | Jarvis parameter expressing the effect of VPD on g_s_ |  | Dimensionless | 8.06 | This work |
| *a_temp_* | Jarvis parameter expressing the effect of temperature on g_s_ |  | °C^-1^ | 0.23 | This work |
| *b_temp_* | Jarvis parameter expressing the effect of temperature on g_s_ |  | Dimensionless | 22.5 | This work |
| *a_psi_* | Jarvis parameter expressing the effect of water potential on g_s_ |  | MPa^-1^ | -8.467 | This work |
| *b_psi_* | Jarvis parameter expressing the effect of water potential on g_s_ |  | Dimensionless | 13.129 | This work |
| *a_par_* | Jarvis parameter expressing the effect of PAR on g_s_ |  |  | 0.005 | This work |
|  |  |  |  |  |  |

**Table S4.** Mean values at harvest for fruit and shoot dry mass, fruit fresh mass, concentrations, and amounts of sucrose, glucose, fructose and sorbitol according to the simulations performed. Significant differences among simulations are indicated and p-values are displayed. Abbreviations: LS = low stress, MS = moderate stress, SS = severe stress, Pn = photosynthesis limiting function, Growth = growth-limiting function.

| Scenario | Dry mass (g) | | Fresh mass (g) | Concentration (g g fresh mass^-1^) | | | | Amount (g C) | | | |
| --- | --- | --- | --- | --- | --- | --- | --- | --- | --- | --- | --- |
|  | Fruit | Leafy shoot | Fruit | Sucrose | Glucose | Fructose | Sorbitol | Sucrose | Glucose | Fructose | Sorbitol |
| Control | 19.35 c | 2.39 a | 117.02 f | 0.038 a | 0.012 a | 0.014 a | 0.012 a | 1.758 d | 0.538 c | 0.597 c | 0.502 d |
| LS | 19.35 c | 2.39 a | 100.91 e | 0.045 b | 0.014 b | 0.016 b | 0.014 b | 1.758 d | 0.538 c | 0.597 c | 0.502 d |
| MS | 19.35 c | 2.39 a | 94.52 d | 0.048 cd | 0.015 c | 0.017 c | 0.015 bc | 1.758 d | 0.538 c | 0.597 c | 0.502 d |
| SS | 19.35 c | 2.39 a | 90.06 d | 0.051 d | 0.016 cd | 0.018 d | 0.016 c | 1.758 d | 0.537 c | 0.597 c | 0.502 d |
| LS/Pn | 18.15 bc | 2.38 a | 93.89 d | 0.044 b | 0.014 b | 0.016 b | 0.014 b | 1.609 c | 0.484 b | 0.538 b | 0.484 cd |
| MS/Pn | 17.56 b | 2.38 a | 84.34 c | 0.047 c | 0.015 c | 0.017 c | 0.015 bc | 1.523 bc | 0.464 b | 0.515 b | 0.462 b |
| SS/Pn | 17.26 b | 2.38 a | 78.51 b | 0.049 d | 0.016 c | 0.018 d | 0.016 c | 1.476 b | 0.455 b | 0.505 b | 0.448 b |
| LS/Growth | 18.80 c | 2.29 a | 97.49 d | 0.044 b | 0.014 b | 0.016 b | 0.014 b | 1.671 cd | 0.513 bc | 0.569 c | 0.490 cd |
| MS/Growth | 17.88 b | 2.24 a | 85.60 cd | 0.046 c | 0.016 c | 0.017 c | 0.014 b | 1.515 b | 0.487 b | 0.541 b | 0.440 ab |
| SS/Growth | 17.70 b | 2.22 a | 80.49 bc | 0.048 cd | 0.017 d | 0.018 d | 0.015 bc | 1.486 b | 0.486 b | 0.539 b | 0.425 ab |
| LS/Pn+Growth | 17.63 b | 2.29 a | 90.61 d | 0.044 b | 0.014 b | 0.015 b | 0.014 b | 1.527 bc | 0.463 b | 0.514 b | 0.469 c |
| MS/Pn+Growth | 16.27 a | 2.24 a | 76.36 b | 0.045 bc | 0.015 c | 0.017 c | 0.015 bc | 1.308 a | 0.427 a | 0.473 a | 0.396 a |
| SS/Pn+Growth | 15.89 a | 2.22 a | 70.44 a | 0.047 c | 0.017 d | 0.018 d | 0.017 c | 1.247 a | 0.419 a | 0.465 a | 0.371 a |
| Factorial analysis | | | | | | | | | | | |
| Irrigation level (I) | < 0.001 | 0.717 | < 0.001 | < 0.001 | < 0.001 | < 0.001 | < 0.001 | < 0.001 | < 0.001 | < 0.001 | < 0.001 |
| Limiting function (LF) | < 0.001 | 0.017 | < 0.001 | < 0.001 | < 0.001 | < 0.001 | < 0.001 | < 0.001 | < 0.001 | < 0.001 | < 0.001 |
| I x LF | 0.030 | 0.999 | 0.003 | < 0.001 | < 0.001 | < 0.001 | < 0.001 | < 0.001 | 0.369 | 0.352 | < 0.001 |

Different letters in the columns indicate significant differences among scenarios at *p* < 0.05.

# Supplementary Figures

# The supplementary material includes four figures. The first two display the within-tree variability of photosynthesis per leaf surface (Supplementary Figure 1) and direct photosynthetically active radiation (Supplementary Figure 2). The third figure shows the evolution of the ratio between fruit dry and fresh masses (Supplementary Figure 3). The fourth figure displays the evolution of the concentration of four sugars in the fruit on a fresh-mass basis (Supplementary Figure 3).

**
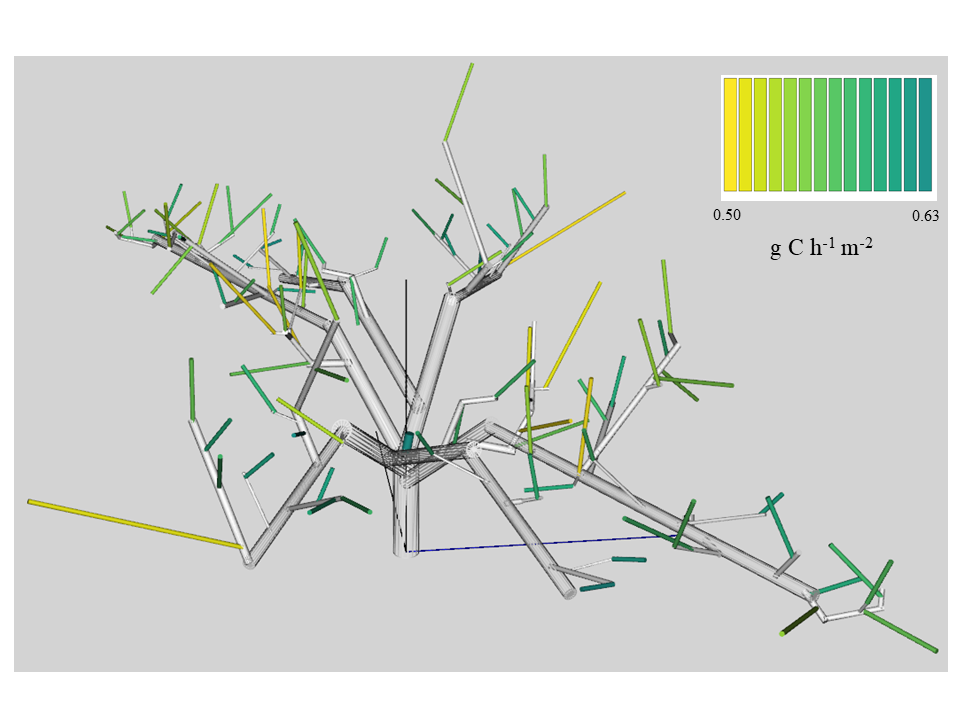
**

**Supplementary Figure 1.** Photosynthesis per leaf surface unit at 14 hours in mid-July (14^th^) of an Elberta peach tree, under the low stress (LS) treatment, simulated by QualiTree. The colors represent the photosynthesis values (gC h^-1^ m^-2^) for each FU.

**
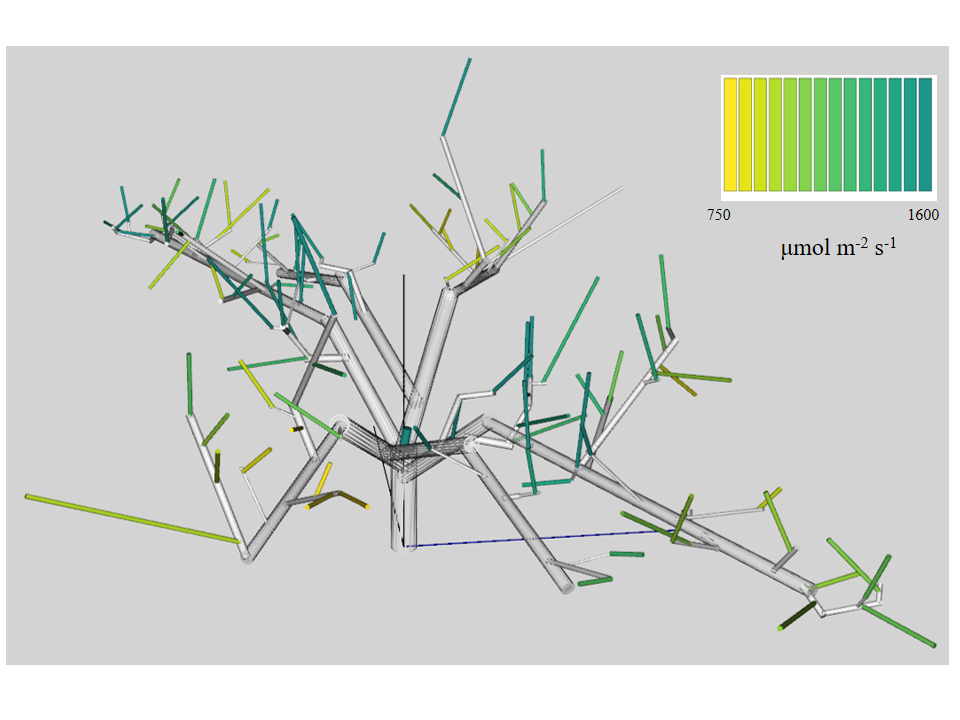
**

**Supplementary Figure 2.** Direct photosynthetically active radiation (PAR) received by an Elberta peach tree, under the low stress (LS) treatment, at 14 hours in mid-July (14^th^), as simulated by QualiTree. The colors represent the PAR values (μmol m^-2^ s^-1^) for each FU.


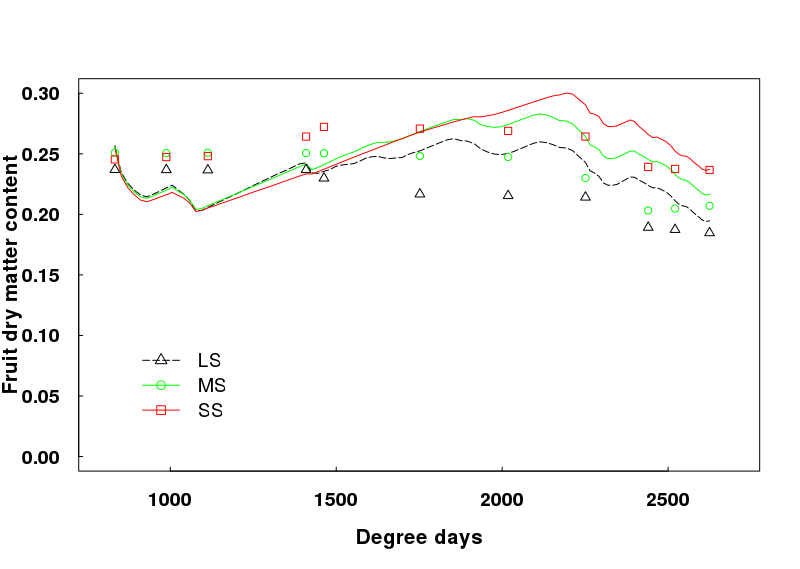


**Supplementary Figure 3.** Evolution of the fruit dry matter content as a function of degree days for the three treatments considered. Observed (points) and simulated (lines) values are shown. Harvest time was 2625 degree-days. Abbreviations: LS = low stress, MS = moderate stress and SS = severe stress.


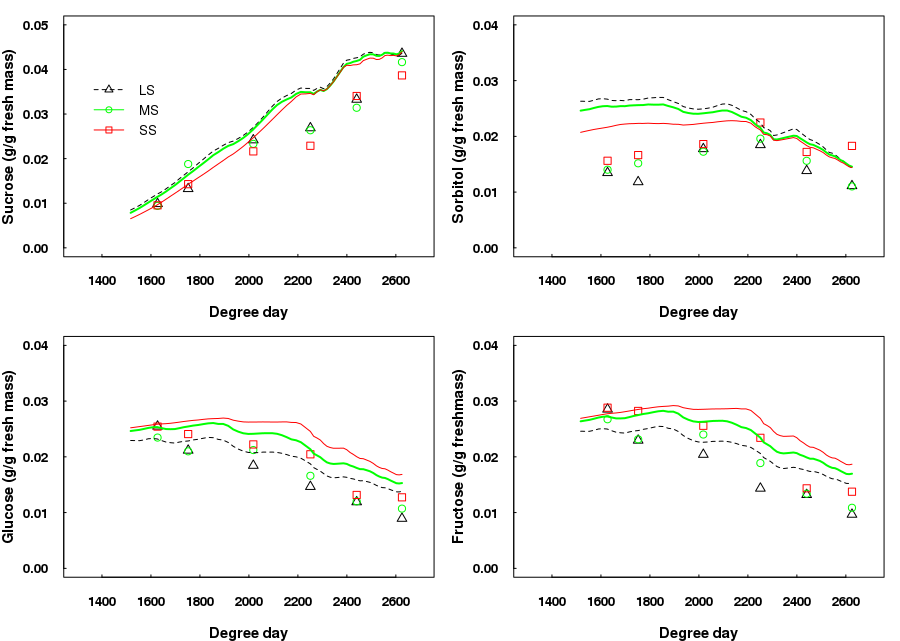


**Supplementary Figure 4.** Test of the model against experimental data for the Elberta trees grown under different irrigation levels in the semi-arid conditions of Iran. Variations of sugar concentrations on a fresh mass basis among monitored shoots as a function of degree days, either observed (points) or simulated (lines). Harvest time was 2625 degree-days. Abbreviations: LS = low stress, MS = moderate stress and SS = severe stress.

**References**

Ashworth, E.N., Stirm, V.E., and Volenec, J.J. (1993). Seasonal variations in soluble sugars and starch within woody stems of *Cornus sericea* L. *Tree Physiol.* 13, 379-388.

Ben Mimoun, M. (1997). Vers la maîtrise de la variabilité des fruits au sein de l'arbre: étude et modélisation de la croissance des pêches (*Prunus persica*) à l'échelle du rameau. Thèse de Doctorat, INA PG, 98 p.

Berman, M.E., and DeJong, T.M. (2003). Seasonal patterns of vegetative growth and competition with reproductive sinks in peach (*Prunus persica*). *J. Horticult. Sci. Biotech.* 78, 303-309.

DeJong, T.M., and Goudriaan, J. (1989). Modeling peach fruit growth and carbohydrate requirements: re-evaluation of the double-sigmoid growth pattern. *J. Am. Soc. Hortic. Sci.* 114, 800-804.

Fishman, S., and Génard, M. (1998). A biophysical model of fruit growth: Simulation of seasonal and diurnal dynamics of mass. *Plant Cell Environ.* 21, 739-752

Génard, M., and Souty, M. (1996). Modeling the peach sugar contents in relation to fruit growth. *J. Am. Soc. Hortic. Sci.* 121, 1122-1131.

Grossman, Y.L., and DeJong, T.M. (1994a). Carbohydrate requirements for dark respiration by peach vegetative organs. *Tree Physiol.* 14, 37-48.

Grossman, Y.L., and DeJong T.M. (1994b). PEACH: A simulation model of reproductive and vegetative growth in peach trees. *Tree Physiol.* 14, 329-345.

Higgins, S.S., Larsen, F.E., Bendel, R.B., Radamaker, G.K., Bassman, J.H., Bidlake, W.R., and Al Wir, A. (1992). Comparative gas exchange characteristics of potted, glass-house-grown almond, apple, fig, grape, olive, peach and Asian pear. *Sci. Hortic.* 52, 313-329.

Hipps, N.A., Pagès, L., Huguet, J.G., and Serra, V. (1995). Influence of controlled water supply on shoot and root development of young peach trees. *Tree Physiol.* 15, 95-103.

Lescourret, F., Ben Mimoun, M., and Génard, M. (1998). A simulation model of growth at the shoot-bearing fruit level. I. Description and parameterization for peach. *Eur. J. Agron.* 9, 173-178.

Lescourret, F., and Génard, M. (2005). A virtual peach fruit model simulating changes in fruit quality during the final stage of fruit growth. *Tree Physiol.* 25, 1303-1315.

Lescourret, F., Moitrier, N., Valsesia, P., and Génard, M. (2011). QualiTree, a virtual fruit tree to study the management of fruit quality. I. Model development. *Trees Struct. Funct.* 25, 519-530.

Mediene, S., Jordan, M.O., Pagès, L., Lebot, J., and Adamowicz, S. (2002). The influence of severe shoot pruning on growth, carbon and nitrogen status in young peach trees (*Prunus persica*). *Tree Physiol.* 22, 1289-1296.

Mirás-Avalos, J.M., Egea, G., Nicolás, E., Génard, M., Vercambre, G., Moitrier, N., Valsesia, P., González-Real, M.M., Bussi, C., and Lescourret, F. (2011). QualiTree, a virtual fruit tree to study the management of fruit quality. II. Parameterisation for peach, analysis of growth-related processes and agronomic scenarios. *Trees Struct. Funct.* 25, 785-799.

Mirás-Avalos, J.M., Alcobendas, R., Alarcón, J.J., Valsesia, P., Génard, M., and Nicolás, E. (2013). Assessment of the water stress effects on peach fruit quality and size using a fruit tree model, QualiTree. *Agric. Water Manage.* 128, 1-12.

Moing, A., and Gaudillère, J.P. (1992). Carbon and nitrogen partitioning in peach/blum grafts. *Tree Physiol.* 10, 81-92.

Pavel, E.W., and DeJong, T.M. (1993). Seasonal CO_2_ exchange patterns of developing peach (*Prunus persica*) fruits in response to temperature, light and CO_2_ concentration. *Physiol. Plant.* 88, 322-330.

Penning de Vries, F.W.T., Jansen, D.M., ten Berge, H.F.M., and Bakema, A. (1989). Simulation of ecophysiological processes of growth in several annual crops. In: Simulation Monographs, vol. 29. Pudoc, Wageningen.

Quilot, B., Génard, M., Kervella, J., and Lescourret, F. (2004). Analysis of genotypic variation in fruit flesh total sugar content via an ecophysiological model applied to peach. *Theor. Appl. Genet.* 109, 440-449.

Rieger, M., and Marra, F. (1994). Responses of young peach trees to root confinement. *J. Am. Soc. Hortic. Sci.* 119, 223-228.

Spann, T.M., Beede, R.H., and DeJong, T.M. (2008). Seasonal carbohydrate storage and mobilization in bearing and non-bearing pistachio (*Pistacia vera*) trees. *Tree Physiol.* 28, 207-213.
